# A Systematic Review of Brainstem Contributions to Autism Spectrum Disorder

**DOI:** 10.3389/fnint.2021.760116

**Published:** 2021-11-01

**Authors:** Ala Seif, Carly Shea, Susanne Schmid, Ryan A. Stevenson

**Affiliations:** ^1^Brain and Mind Institute, University of Western Ontario, London, ON, Canada; ^2^Department of Anatomy and Cell Biology, Schulich School of Medicine and Dentistry, University of Western Ontario, London, ON, Canada; ^3^Department of Psychology, University of Western Ontario, London, ON, Canada

**Keywords:** autism spectrum disorder, brainstem, development, sensory, olivary complex, auditory, systematic review

## Abstract

Autism spectrum disorder (ASD) is a neurodevelopmental disorder that affects one in 66 children in Canada. The contributions of changes in the cortex and cerebellum to autism have been studied for decades. However, our understanding of brainstem contributions has only started to emerge more recently. Disruptions of sensory processing, startle response, sensory filtering, sensorimotor gating, multisensory integration and sleep are all features of ASD and are processes in which the brainstem is involved. In addition, preliminary research into brainstem contribution emphasizes the importance of the developmental timeline rather than just the mature brainstem. Therefore, the purpose of this systematic review is to compile histological, behavioral, neuroimaging, and electrophysiological evidence from human and animal studies about brainstem contributions and their functional implications in autism. Moreover, due to the developmental nature of autism, the review pays attention to the atypical brainstem development and compares findings based on age. Overall, there is evidence of an important role of brainstem disruptions in ASD, but there is still the need to examine the brainstem across the life span, from infancy to adulthood which could lead the way for early diagnosis and possibly treatment of ASD.

## Introduction

Autism spectrum disorder (ASD) is a neurodevelopmental disorder with a high prevalence of 1 in 66 in Canada and it disproportionately affects male with prevalence of 1 in 42 (Ofner et al., 2018). A range of core ASD symptoms, such as impairments in social communication skills combined with restricted and repeated behaviors and interests, as well as motor stereotypies, could be related to sensory disruptions (Sinclair et al., 2017). Autistic individuals also avoid sensory stimuli and/or display sensory seeking behavior (Sinclair et al., 2017). Moreover, disruptions of sensory processing, startle response, sensory filtering, sensorimotor gating, multisensory integration, and sleep are all features of ASD and are processes that the brainstem is crucially involved in.

The brainstem is the structure that connects the cerebrum to the spinal cord and cerebellum. It consists of three main components in ascending order: medulla oblongata, pons, and the midbrain (Moller, 2006). It is a conduit for the ascending and descending pathways between the cerebellum and spinal cord, and it houses the cranial nerves and integrated vital systems. Autistic individuals often experience symptoms associated with auditory brainstem abnormalities such as difficulty with auditory temporal processing while having normal hearing threshold, impaired sound localization, poor speech recognition in noise and abnormal sound sensitivity (Pillion et al., 2018). The auditory brainstem pathway includes the cochlear nucleus (CN), superior olivary complex (SOC), and inferior colliculus which are in pairs on either side of the brainstem and connected by afferent relay pathways, including the lateral lemniscus. The CN receives input from the peripheral auditory system and is located at the connection between the medulla and the pons. The CN's main function is to maintain the frequency information extracted by the cochlea in the inner ear. It then projects bilaterally to the SOC, where binaural sound cues are processed for sound localization. The olivocochlear bundle, which projects from the SOC, serves to protect the inner ear from loud sounds and high levels of background noise (Moller, 2006).

The pathogenesis of ASD has long been studied, but no definitive conclusion has been reached. The alterations of cortex and cerebellum are investigated regularly due to their more obvious involvement in the cognitive symptoms of autism such as social difficulties and language delay. What is more often overlooked is the cascading effect of abnormal brainstem development and/or delays in brainstem processing on higher brain centers. Studying the potential origin of autism symptoms in the brainstem is important for early diagnosis and intervention.

Our understanding of brainstem contributions to autism has only started to emerge. Research involving the brainstem has been relatively slow due to technological difficulties. Its small size, functional diversity, and anatomy makes the brainstem less accessible for studying than the cortex. There is, however, a growing body of evidence regarding its contributions to autism. Therefore, this systematic review compiles research investigating the involvement of the brainstem in the pathogenesis of ASD. This paper includes studies examining histological, neuroimaging, and electrophysiological evidence from human and animal studies about brainstem contributions and their functional implications in autism. Moreover, we compared papers with distinct age ranges for an understanding of developmental delay implication on ASD. This is especially important due to the developmental nature of ASD.

## Methods

### Study Design

A systematic review was chosen due to its systematic approach in identifying and summarizing all the work in this field. This review follows the guidelines set by the Joanna Briggs Institute, according to the Manual for Evidence Synthesis (Aromataris and Munn, 2020), the Peer Review of Electronic Search Strategies Guidelines (McGowan et al., 2016), and the Preferred Reporting Items for Systematic Reviews and Meta-Analysis (Shamseer et al., 2015). This project was pre-registered with the Open Science Foundation (osf.io/2hd6m).

### Eligibility Criteria

The eligibility criteria for the included work is based on the population, exposure, comparison and outcome (PICO) framework (Aromataris and Munn, 2020). The population is defined as anyone with a clinical diagnosis of ASD including autism, Asperger syndrome, Atypical autism and the equivalents in Diagnostic and Statistical Manual of Mental Disorders (DSM-III; American Psychiatric Association, 1980), DSM-IV (American Psychiatric Association, 1994), and DSM-V (American Psychiatric Association, 2013) classification systems and/or with confirmation of diagnosis using Autism Diagnostic Interview-Revised (ADI-R; Rutter et al., 2003) or Autism Diagnostic Observation Schedule (ADOS; Lord et al., 1999). Moreover, participants with comorbidities are also included due to their high prevalence in autism. The study population also included animal models of autism. The intervention/methodologies are the application of histological, neuroimaging, or electrophysiological experiments designed to study the involvement of brainstem in autism. The comparison is to healthy controls and the outcome is the experimental results. Case studies and case series were excluded, and all studies had to be controlled. Finally, we did not limit the search by a start date to ensure the comprehensiveness of this review. We restricted the review to English, peer-reviewed and primary research publications.

### Search Strategy

#### Information Sources

The search strategy was reviewed by a library scientist (Meagan Stanley) and reviewed by two independent researchers (R.S, S.S). Initial search terms were piloted in two databases, MEDLINE and EMBASE, and titles and abstracts were screened for additional search terms. We applied our search within MEDLINE, EMBASE, and PsychInfo through the Ovid interface, as well as CINAHL via the EBSCO interface, SCOPUS, and Cochrane library. We used appropriate subject headings for each database and all key words synonymous with “autism spectrum disorder” and “brainstem.” The search strategy is in Table 1. The preliminary search was conducted on October 25th, 2020 and the final search was conducted on October 28th, 2020.

**Table 1:** Search strategy used to search on all databases.

	**Concept 1**	**Concept 2**
Key concepts	Autism	Brainstem
Free text terms/natural language terms (synonyms, UK/US terminology, medical/laymen's terms, acronyms/abbreviations, more narrow search terms)	• autism • autistic • asperger* • PDD-NOS • Kanner syndrome • Kanner's syndrome • Kanner's syndrome • pervasive developmental • PDD-NOS	• brain stem • brain stems • brainstem • brainstems • pons • pon • ponte • pontes • pontine • pontis • Olivary • Olive brain • oliva brain • olivae • olivaris • olive nucleus • midbrain • mid brain • midbrains • mesencephalon • mesencephalons • mesencephalic • mesencephali • colliculus • colliculi • quadrageminal • quadrigeminal • thalamencephalon • thalamencephalons • thalamus • thalamic • neothalamus • thalami • cuneiform nucleus • formatio reticulari • formatio reticularis • reticular formation • reticular formations • reticular substance • reticular system • substantia reticulari
Controlled vocabulary terms/Subject terms (MeSH terms, Emtree terms) *Consider: explode, major headings, subheadings*	• Child Development Disorders, Pervasive • Asperger Syndrome • Autistic Disorder • Autism Spectrum Disorder • autism	• Reticular Formation • Midbrain Reticular Formation • Thalamus • thalamus reticular nucleus • Thalamic Nuclei • Superior Colliculi • Inferior Colliculi • Mesencephalon • Pons • pontine tegmentum • ventral pons • pons reticular formation • pons angle • olivary nucleus • olivary body • Brain Stem

#### Selection and Sources of Evidence

Two independent reviewers (A.S and C.S or R.S) screened the titles and abstracts of every unique article for inclusion eligibility through the Covidence software. Subsequently, one reviewer conducted the full-text screening to ensure eligibility criteria is met. Extraction was completed by one reviewer (A.S).

#### Quality Assessment

The quality assessment of the publications included was made based on guidelines set by the Joanna Briggs Institute, according to the Manual for Evidence Synthesis (Aromataris and Munn, 2020). The checklist for each study was as below.

1. Were the groups comparable other than the presence of disease in cases or the absence of disease in controls?
2. Were cases and controls matched appropriately?
3. Were the same criteria used for identification of cases and controls?
4. Was exposure measured in a standard, valid and reliable way?
5. Was exposure measured in the same way for cases and controls?
6. Were confounding factors identified?
7. Were strategies to deal with confounding factors stated?
8. Were outcomes assessed in a standard, valid and reliable way for cases and controls?
9. Was the exposure period of interest long enough to be meaningful?
10. Was appropriate statistical analysis used?

## Results

**Figure 1:**
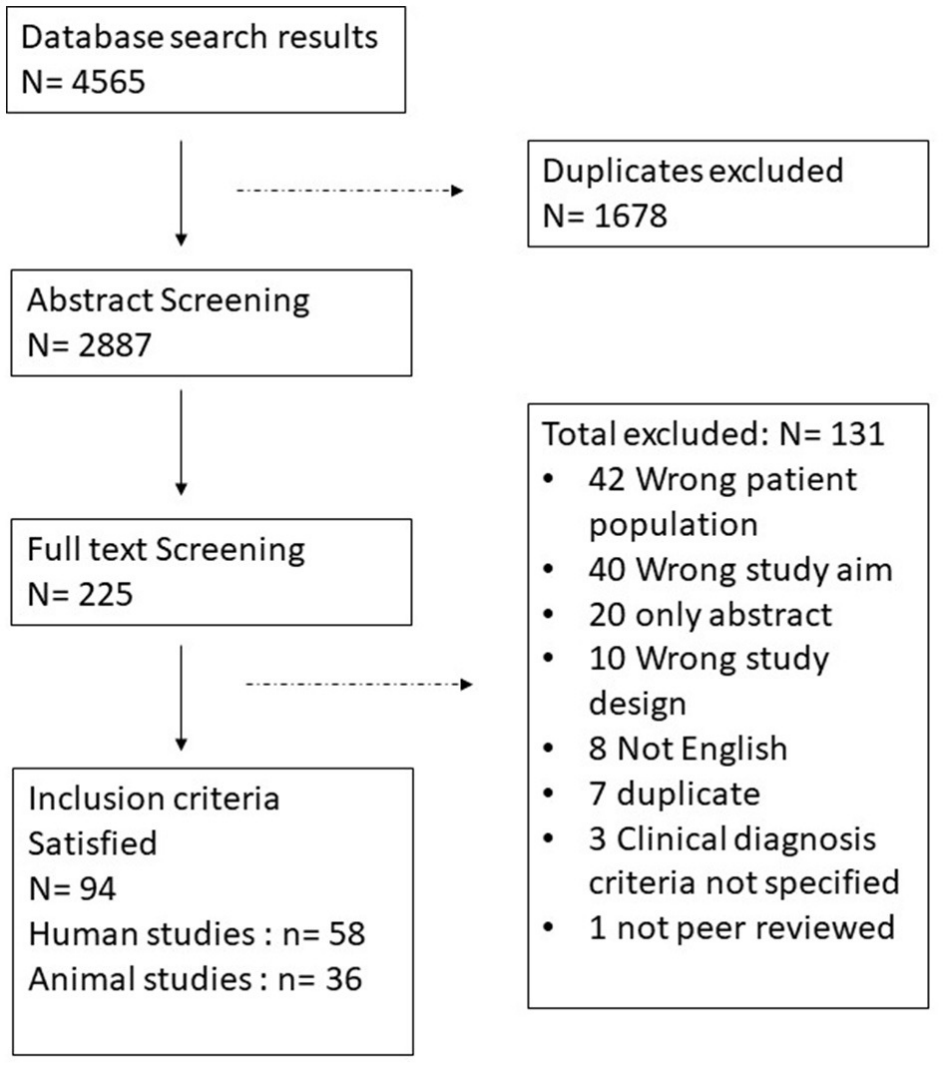
PRISMA diagram, identification and selection process of the papers.

The search identified 4,565 references, of which 1,678 were duplicates. The 2,887 unique studies were screened based on their title and abstract which narrowed them down to 225 papers for full text review. Afterwards 94 studies were accepted to be included of which 58 are human studies and 36 are animal studies (Figure 1: PRISMA Diagram). All included studies fulfilled at least 6 of the quality assessment criteria. Supplementary Table 1 summarizes all human studies and Supplementary Table 2 summarizes all animal studies and includes their quality scores.

### Brainstem Morphology Studies

#### Post-mortem

The morphological studies based on the post-mortem brain are relatively few, possibly due to the difficulty in obtaining brain tissue. Most of the studies in this are led by Kulesza et al. using brain tissue specimens obtained from the Autism Brain Net previously known as Autism Tissue program (Kulesza and Mangunay, 2008; Kulesza et al., 2011; Mansour and Kulesza, 2020). Kulesza et al., investigated the disruption of the cytoarchitecture in the lower auditory brainstem, which is the superior olivary complex (SOC) and cochlear nucleus (CN). The SOC neurons have altered cell body morphology and reduced neuronal numbers in the autistic brains (Kulesza et al., 2011). Moreover, in a three dimensional reconstruction of SOC made using Amira software, the overall volume of SOC nuclei of autistic individuals were significantly smaller than controls (Mansour and Kulesza, 2020).

The medial superior olive (MSO) nuclei were the most constantly and severely malformed nuclei (Kulesza and Mangunay, 2008; Kulesza et al., 2011). They also occupied significantly smaller volumes in autistic brains compared to controls, as seen in a three-dimensional reconstruction of the SOC. MSO nuclei were significantly smaller than the control in terms of cell body area, perimeter and major axis (Kulesza and Mangunay, 2008). In addition, the neurons were significantly rounder (Kulesza and Mangunay, 2008). However, there was a significant variance in the angle measurements indicating that the orientation of MSO in the autistic group was more heterogenous than the control.

The number of lateral superior olive (LSO) cell nuclei are significantly less in autistic brains compared to control. The LSO neurons occupy significantly smaller volume (Mansour and Kulesza, 2020) and are also significantly smaller in area and more round in autistic brains (Kulesza et al., 2011). There are differences in ratio of the oval to the fusiform to the stellate medial nuclei of the trapezoid body (MNTB) neurons in autism. The number of MNTB neurons is significantly less and the neurons have a trend of being smaller (Kulesza et al., 2011) a finding corroborated via three-dimensional reconstruction (Mansour and Kulesza, 2020). Moreover, they are less round and of different orientation (Kulesza et al., 2011). The superior paraolivary nucleus (SPON) neurons occupy a significantly smaller volume, are less in number, and rounder in autistic brains (Kulesza et al., 2011). Ventral nucleus of the trapezoid body (VNTB) neurons are rounder in ASD (Kulesza et al., 2011). Lateral nucleus of the trapezoid body neurons are significantly fewer (Kulesza et al., 2011), smaller (Kulesza et al., 2011; Mansour and Kulesza, 2020), and rounder in the autistic brain (Kulesza et al., 2011). Finally, the overall volume of the SOC is consistent across brain weights and changes in the size of the SOC, and its constituent nuclei in ASD are not likely due to changes in total brain weight (Mansour and Kulesza, 2020). SOC nuclei have a role in sound localization and coding of sound temporal features (Mansour and Kulesza, 2020), therefore those alterations could have a role in the auditory dysfunction experienced in ASD.

Wegiel et al. (2014) examined the percentage of nucleus volume in a neuronal cell in different brain regions including inferior olive and substantia nigra in a post-mortem study. They divided the participants into 3 different groups based on age, 4–8, 11–23, and 29–64 years. Deficits in volume of neuronal nucleus were significant only for the age group 4–8 years (Wegiel et al., 2014). The deficit was moderate (<20%) in the inferior olive and mild (<10%) in substantia nigra. The neuronal cytoplasm volume was measured as total neuron volume minus neuronal nucleus volume. In ASD, there is a deficit of 4% of neuronal cytoplasm volume in the substantia nigra (Wegiel et al., 2014). In addition, the trajectory of neuronal nucleus volume in ASD was opposite to the control trajectory. The autistic cohort had a significant increase in at least one of the older groups while a distinctive feature of control was a decrease in neuronal nucleus volumes in both older groups (Wegiel et al., 2014). A different study quantified the oxytocin receptor and the structurally related vasopressin 1a receptor in the superior colliculi in post-mortem specimens of autistic and neurotypical individuals and found no difference (Freeman et al., 2018).

A study used immunocytochemistry to compare angiogenesis in post-mortem brains of 10 autistic young adults and their age matched controls. It reported increased and prolonged neural development in the brainstem including midbrain and pons (Azmitia et al., 2016).

The takeaway of post-mortem studies is that there is an age dependent (Wegiel et al., 2014) malformation in the shapes of the different olivary complex cells (Kulesza and Mangunay, 2008; Kulesza et al., 2011; Wegiel et al., 2014; Mansour and Kulesza, 2020) and substantia nigra (Wegiel et al., 2014). However, it is important to keep in mind that all post-mortem studies are based on small samples.

#### Neuroimaging

Analysis of brainstem size and its components (pons, medulla, midbrain) using MRI scans has mixed results. The most common result is a reduction in brainstem size. One of the earliest studies suggested a trend in brainstem area reduction (Hashimoto et al., 1989). Another early study, found that the brainstem and pons of autistic individuals was significantly smaller than in controls (Gaffney et al., 1988). Recent studies confirmed these results. A significant reduction in total brainstem size has been consistently observed (Gaffney et al., 1988; Hashimoto et al., 1995; Herbert et al., 2003; Fredo et al., 2014). In one study of 20 adult autistic men and their age, gender and IQ matched controls, total brainstem volume was reduced, however, it was proportional to total brain volume and volume of other regional areas (Herbert et al., 2003). Two studies reported a reduction in brainstem gray matter (Jou et al., 2009; Toal et al., 2010). The first, a study of 22 autistic male children, showed significant reduction in gray matter before and after controlling for total brain volume (Jou et al., 2009) while the second, a study of 39 adults males diagnosed with Asperger's syndrome (Toal et al., 2010), also showed gray matter reduction when compared with age and gender matched controls. A study using PET scans to examine serotonin transporter availability in the gray matter of 15 autistic adults and their matched controls (age, gender, IQ) exhibited significant reduction in serotonin transporter availability in brainstem gray matter (Andersson et al., 2020). Three studies that used voxel based morphometry to investigate white matter reported brainstem white matter reduction (Craig et al., 2007; Toal et al., 2010; Hanaie et al., 2016). One of the studies investigated the white matter in autistic women and concluded that they had a smaller density of white matter in pons (Craig et al., 2007). While another study reported that white matter in the regions corresponding to parts of the central tegmental tract/medial lemniscus (CTT/ML) were significantly smaller in ASD group (Hanaie et al., 2016).

Hashimoto et al. conducted 8 studies investigating the brainstem structures by analyzing MRI scans. One of the biggest studies was with a sample of 102 ASD participants in the age range of 3 months to 20 years along with 112 age- and gender-matched controls (Hashimoto et al., 1995). The results reported that the brainstem size and the size of its three components (pons, midbrain, and medulla oblongata) increased with development and revealed a statistically significant correlation coefficient with age for both groups but the area of the brainstem in the autistic group was significantly smaller than those in the control group across all ages (Hashimoto et al., 1995). A study reported a significantly smaller midbrain, pons and medulla oblongata for the autistic group (Hashimoto et al., 1993b) while another study reported the significance for smaller size only in the midbrain and medulla oblongata when the groups were IQ matched (Hashimoto et al., 1993a). In a study in which autistic participants with intellectual disability were compared to non-autistic participants with intellectual disability, there was no absolute difference in brainstem area but there was a significant reduction in ratio of midbrain and medulla to posterior fossa (Hashimoto et al., 1992a). Moreover, when autistic participants were grouped into either a heterogenous IQ group, low IQ group (IQ < 80) or high IQ group (IQ > 80), all groups indicated that MRI brainstem width in autistic children differs from that in the control group. Brainstem width of the ASD group was smaller than the control and this difference tends to be exacerbated in the low IQ group (Hashimoto et al., 1992b). The maximum width in the middle portion of the pons was significantly smaller for the autistic heterogenous IQ group and autistic low IQ group compared to the controls (Hashimoto et al., 1992b).

However, another study with a much smaller sample size compared to previous studies had an opposing outcome of increased brainstem volume in autism when comparing 6 autistic children with mean age 53 ± 16 months to 38 controls with matching age, gender, and intellectual functioning (Bosco et al., 2019). A study that compared the pons size of 14 autistic participants to 2 control groups either age, sex and IQ matched or age, sex and socioeconomic status matched found no group difference after correcting for mid-sagittal brain area (Piven et al., 1992). Two other studies investigating the brainstem area reported no significant difference between autistic participants and healthy controls (Garber and Ritvo, 1992; Hardan et al., 2001).

Another group used high angular resolution diffusion-weighted imaging and functional MRI data to examine structural and functional connectivity of the mesolimbic reward pathway (Supekar et al., 2018). They identified the nucleus accumbens and the ventral tegmental area (VTA) white matter tract and found structural aberrations in these tracts in two cohorts of autistic children (Supekar et al., 2018). Moreover, they showed that structural aberrations are accompanied by aberrant functional interactions between nucleus accumbens and VTA in response to social stimuli (Supekar et al., 2018).

Two studies aimed to study textural features of the brainstem, which provide a complementary basis for volumetric morphometric analysis by summarizing distributions of localized image measurements (Chaddad et al., 2017). One study observed that the mean entropy values, which is the distribution of pixels values over intensity levels of a MRI scan, obtained from the subcortical regions are significantly higher in 30 autistic subjects (Jac Fredo et al., 2015) while the second study with 575 ASD participants concluded no significant textural feature differences in the brainstem (Chaddad et al., 2017).

In summary the brainstem of autistic individuals is likely smaller in size than healthy controls as concluded by most studies (Gaffney et al., 1988; Hashimoto et al., 1989, 1992a,b, 1993a,b, 1995; Herbert et al., 2003; Craig et al., 2007; Jou et al., 2009; Toal et al., 2010; Fredo et al., 2014; Hanaie et al., 2016; Andersson et al., 2020). The reduction is also observed in the brainstem white matter (Craig et al., 2007; Toal et al., 2010; Hanaie et al., 2016) and gray matter (Jou et al., 2009; Toal et al., 2010). In studies that investigated specific brainstem components, a reduction is seen in the medulla (Hashimoto et al., 1992a,b, 1993a,b, 1995), the midbrain (Hashimoto et al., 1992a,b, 1993a,b, 1995) and the pons (Hashimoto et al., 1992b, 1993b, 1995). These results go hand in hand with post-mortem studies indicating abnormalities in the overall size of brainstem and malformation of component nuclei of it.

#### Neuroimaging Relating to Behavior

The relationship between brainstem anatomy and sensory-motor function has also been evaluated in autism. A group investigated the relationship between brainstem gray matter volume and sensory sensitivity as measured by the Sensory Profile Questionnaire (SPQ) and it observed a significant positive correlation between oral sensory sensitivity factor and brainstem gray matter (Jou et al., 2009). Another group investigated if atypical white matter microstructure in the brain mediated the relationship between motor skills and ASD symptom severity. Fractional anisotropy of the brainstem corticospinal tract predicted both grip strength and autism symptom severity and mediated the relationship between the two (Travers et al., 2015). It suggested that brainstem white matter might contribute to autism symptoms and grip strength in ASD (Travers et al., 2015). Another group aimed to relate brainstem white matter to motor performance in autism using Movement Assessment Battery for Children 2 (M-ABC 2) in which higher scores are indicative of better motor performance (Hanaie et al., 2016). The study reported a significant positive correlation between the total test score on the M-ABC 2 and the volume of brainstem white matter (Hanaie et al., 2016).

A study investigated the correlation between language development and neuroanatomy of ASD participants with and without a language delay and neurotypicals (Lai et al., 2014). Neuroanatomy was assessed by MRI images and language development was measured by verbal IQ through a word generativity test using the F-A-S task and a phonological memory test using the non-word repetition task (Lai et al., 2014). Language delay was associated with larger total gray matter volume and larger relative volume of the pons and medulla oblongata in adulthood (Lai et al., 2014).

Another study utilized fMRI to examine the effect of constraining gaze in the eye-region on activation of the subcortical system, specifically the superior colliculus in the brainstem. Participants looked at facial emotional stimuli by either free-viewing or by being restricted to eye-region conditions (Hadjikhani et al., 2017). ASD and controls had similar activation patterns in free viewing but the ASD group had higher superior colliculus activation in the constrained to look in the eyes condition (Hadjikhani et al., 2017). Additionally, there was a positive correlation between autism symptom severity and subcortical system activation for stimuli of fear and neutral faces, in the free viewing condition (Hadjikhani et al., 2017).

Finally, a study used imaging-genetics data from a discovery sample of 27,034 individuals and identified 45 brainstem-associated genetic loci, including the first linked to midbrain, pons, and medulla oblongata volumes, and mapped them to 305 genes (Elvsåshagen et al., 2020). Of those genetic loci, 9 were jointly associated with the brainstem volumes and autism (Elvsåshagen et al., 2020). Notably, the shared genetic loci exhibited a mixed pattern of allelic effect directions such as associations with both larger and smaller brainstem volumes (Elvsåshagen et al., 2020).

### Functional Studies

#### Auditory Brainstem Response (ABR)

**Figure 2:**
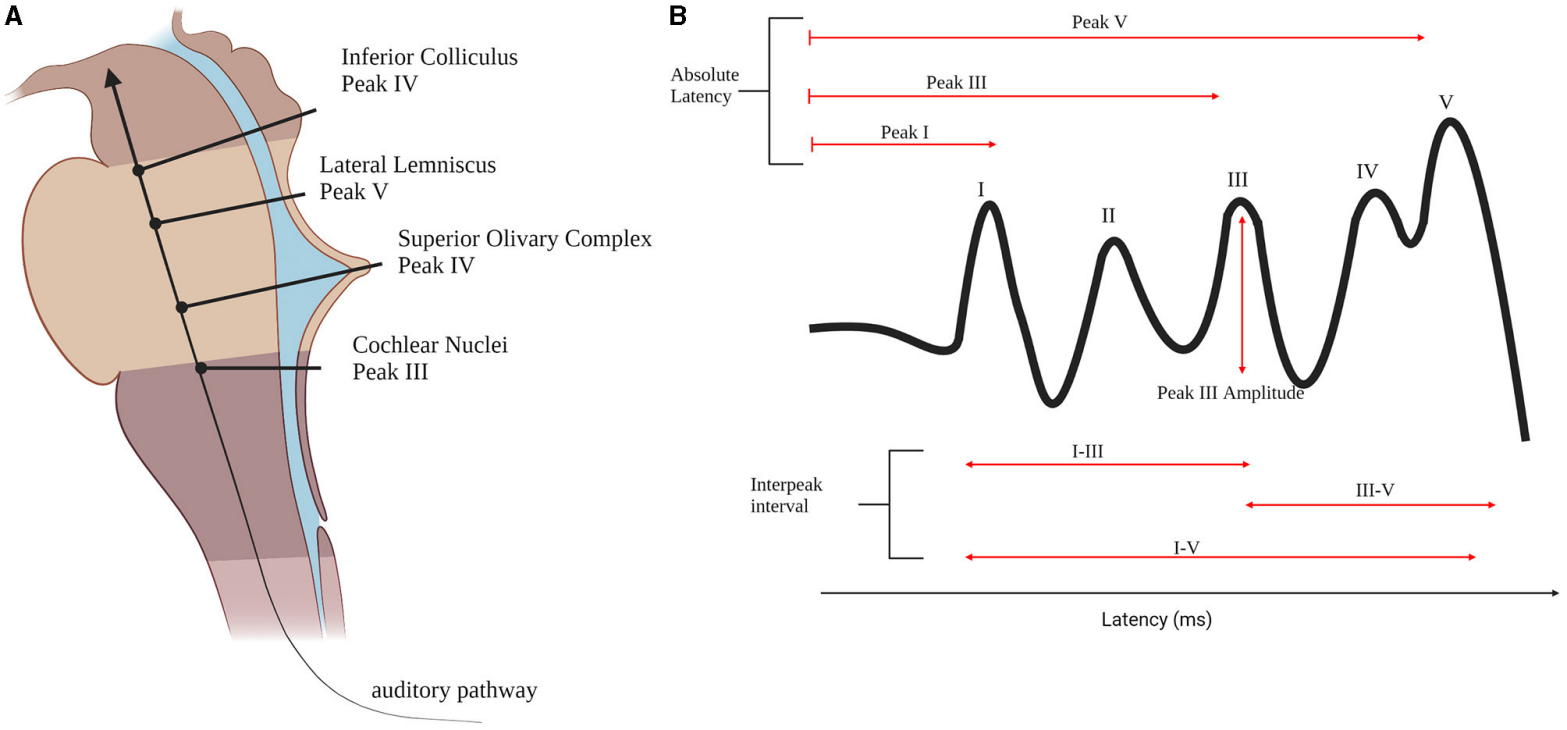
Brainstem auditory pathway. **(A)** The brainstem auditory pathway and the corresponding ABR peaks generators in the brainstem. **(B)** An ABR wave and the measures found to be different between autistic individuals and controls. Created with bioRender.com.

ABRs are auditory evoked potentials measured at the scalp that are used clinically to assess the functional integrity of the auditory pathway. ABRs consist of five distinct positive peaks during the first 10 ms following the presentation of a sound (Moller, 2006). Peak I and peak II are generated by the auditory nerve while the other peaks are generated by the contributions of different anatomical structures (Moller, 2006). Peak III is generated by the CN. The SOC is suggested to be the generator of peak IV (Moller, 2006). The peak V tip is generated by the lateral lemniscus (LL) and it terminates on the inferior colliculus (IC) (Figure 2A; Moller, 2006).

The most studied aspects of ABR waveforms are the absolute latencies of the peaks and the interpeak latencies, while few papers examine peak amplitudes. Peak I latency is found to be prolonged in ASD (Tanguay et al., 1982; Rosenhall et al., 2003; Magliaro et al., 2010; Azouz et al., 2014; Ververi et al., 2015) when compared to controls. This suggests the presence of very early differences in auditory processing in autism, even as early as the auditory nerve (Figure 2B). Peak III latency is also found to be delayed (Magliaro et al., 2010) and prolonged (Tanguay et al., 1982) as well as peak I- III inter peak latency (Maziade et al., 2000; Magliaro et al., 2010) possibly due to alterations at the CN (Figure 2B). In addition, peak I-III IPL is found to be prolonged in autistic probands and their first-degree relatives (Maziade et al., 2000). Peak V is also found to be prolonged in ASD groups when compared to healthy controls (Rosenhall et al., 2003; Russo et al., 2008; Azouz et al., 2014; Ververi et al., 2015) which is generated by LL and IC (Figure 2B), but could potentially be due to the cascading effect of previous locations along the primary auditory pathway such as SOC. As previously discussed, the morphology of SOC is found to be altered in the post-mortem brains of autistic individuals (Kulesza and Mangunay, 2008; Kulesza et al., 2011; Wegiel et al., 2014; Mansour and Kulesza, 2020). Moreover, abnormalities in peak III-V IPL (Figure 2B) are found (Rosenhall et al., 2003; Tas et al., 2007; Azouz et al., 2014; Jones et al., 2020; Kamita et al., 2020). The most common ABR finding is that the wave is prolonged in ASD (Rosenhall et al., 2003; Tas et al., 2007; Azouz et al., 2014; Kamita et al., 2020). IPL of peak I-V differences are found as well (Figure 2B; Ververi et al., 2015; Jones et al., 2020).

Amplitudes are not commonly examined, and results are inconsistent. A group found peak III amplitude to be lower for ASD when a forward masking condition was applied (Figure 2B; Källstrand et al., 2010) while two others found it to be higher (Ververi et al., 2015; Claesdotter-Knutsson et al., 2019). Another study found that the ASD group had higher correlation between right and left ears compared to the typically developing participants (Claesdotter-Knutsson et al., 2019) which could be related to difficulties in the processing of everyday sounds.

ABRs are also used as a conventional hearing test for newborns. Some studies have found abnormalities in click evoked ABR of newborns that were later diagnosed with autism (Cohen et al., 2013; Miron et al., 2016, 2021). They had significant prolongations of their ABR phase and V-negative latency compared to the newborns that weren't diagnosed with ASD (Miron et al., 2021). A longitudinal study with a year time difference found typically developed toddlers did not have a change in their ABR while autistic toddlers had a shortening in peak V latency and peaks I-V IPL at T2 (Li et al., 2020). Regardless of the change within the two timepoints, autistic toddlers still had longer latencies of peak III and peak V, and longer IPL of peaks I-III and peaks I-V at both timepoints (Li et al., 2020). Nonetheless a study examining the differences between ASD and typically developing controls found no differences (Tharpe et al., 2006).

Speech evoked ABR abnormalities were also identified. Autistic children exhibited deficits in both the neural synchrony (timing; Russo et al., 2009) and phase locking (frequency encoding) of speech sounds (Russo et al., 2008, 2009). They also exhibited reduced magnitude and fidelity of speech evoked ABR and excessive degradation of responses by background noise in comparison to typically developing controls (Russo et al., 2009). A study examined speech evoked ABR at two different timepoints with an average 10 months between them for preschool children. At T1, the wave V and A latencies were prolonged, whereas the wave E amplitude was decreased and the wave F latency prolonged at T2 (Chen et al., 2019). Between the two recordings, the wave V latency was shortened, and amplitudes of wave A and C increased (Chen et al., 2019). Whereas, another study found longer wave V for school aged ASD children (Kamita et al., 2020). A study concluded that latencies of all speech-evoked ABR waves and V-A complex duration are longer in the ASD group compared to healthy controls (Ramezani et al., 2019). Other studies found differences in specific waves such as latencies in waves C, D, E, F (El Shennawy et al., 2014), and wave O (El Shennawy et al., 2014; Jones et al., 2020). Moreover, a difference in amplitude between the ASD and the control groups is observed in waves C, D, E, F, and O (El Shennawy et al., 2014).

The results of ABR are less consistent in terms of how the waves of autistic individuals are different than control but the general trend indicates some form of abnormality (Tanguay et al., 1982; Maziade et al., 2000; Rosenhall et al., 2003; Tas et al., 2007; Russo et al., 2008, 2009; Källstrand et al., 2010; Magliaro et al., 2010; Azouz et al., 2014; El Shennawy et al., 2014; Wegiel et al., 2014; Ververi et al., 2015; Miron et al., 2016, 2021; Chen et al., 2019; Claesdotter-Knutsson et al., 2019; Ramezani et al., 2019; Jones et al., 2020; Kamita et al., 2020; Li et al., 2020).

#### Pupillometry and Eye Tracking

A study utilized changes in pupil size to examine whether autistic individuals exhibit differences in phasic locus coeruleus (LC) activity compared with typically developing controls under different attentional demands. The phasic pupillary response is an indication of LC activity because it is specifically associated with LC-mediated processing and allows for between group comparisons not confounded by unrelated individual differences in pupil size oydistractor tones. The results indicated that under tightly controlled conditions, task-evoked pupil responses are lower in ASD group than in controls, but only in the presence of task-irrelevant stimuli (Granovetter et al., 2019). This suggests that autistic individuals experience atypical modulation of LC activity in accordance with changes in attentional demands, offering a mechanistic account for attentional atypicality in ASD (Granovetter et al., 2019).

A study that utilized eye control to study attentional demands had participants make saccades to peripheral targets while recording their eye movement using EOG (Schmitt et al., 2014). The recordings of the autistic participants had reduced accuracy, elevated variability in accuracy across trials, and reduced peak velocity and prolonged duration (Schmitt et al., 2014). The saccades took longer to reach peak velocity but had no change in the duration of saccade deceleration (Schmitt et al., 2014). Defined brainstem circuits are implicated in the control of saccadic eye movement, therefore this suggested reduced excitatory activity in the burst cells of the pons (Schmitt et al., 2014).

#### Cardiovascular Autonomic Monitoring System

One study aimed to measure baseline cardiovascular autonomic function in autistic children using the NeuroScope, a device that can measure this brainstem function in real-time. They measured resting cardiac vagal tone (CVT), cardiac sensitivity to baroreflex (CSB), mean arterial blood pressure (MAP), diastolic blood pressure (DBP), systolic blood pressure (SBP) and heart rate (HR) for three groups of children (Ming et al., 2005). The groups consisted of autistic children without autonomic abnormality symptoms, autistic children with autonomic abnormalities and age matched healthy controls. The CVT and CSB were significantly lower in association with a significant elevation in HR, MAP and DBP in all autistic children compared with the healthy controls (Ming et al., 2005). Moreover, the levels of CVT and CSB were lower in the symptomatic than in the asymptomatic group. These results suggest that there is low baseline cardiac parasympathetic activity with evidence of elevated sympathetic tone in autistic children regardless of having symptoms or signs of autonomic abnormalities (Ming et al., 2005).

### Animal Studies

Autism is a complicated condition diagnosed on a series of behavioral characteristics that are, in some cases, uniquely human. Due to this complicated nature of autism, there is no single animal model that can represent the diagnosis in its entirety. Instead, different animal models of autism reflect particular symptoms associated with autism. Therefore, there are not animal models that are more or less valid representations of autism as a whole, but rather the choice of animal model used should reflect its ability to mirror the particular symptom or set of symptoms being studied (Möhrle et al., 2020). The animal models included in this review either genetically or environmentally induce such autism core symptoms and are all validated in this respect.

#### *Fmr1* Knockout Model

Fragile X syndrome (FXS) is caused by loss of functional expression of Fmr1 gene and it is the most prevalent single-gene cause of ASD (Ascano et al., 2012). It results from an expansion of the CGG repeats in the promoter region of the *FMR1* gene, which reduces the amount of fragile X mental retardation protein (FMRP) produced (Ascano et al., 2012). FMRP acts as a modulator of mRNA translation and has numerous target genes (Ascano et al., 2012). A study investigated the molecular role of FMRP in the avian nucleus laminaris (NL) which is a brainstem nucleus necessary for binaural processing by performing proteomic analysis of NL (Sakano et al., 2017). They identified 94 proteins that are a potential FMRP target (Sakano et al., 2017). These proteins are enriched in pathways involved in cellular growth, cellular trafficking, and transmembrane transport (Sakano et al., 2017). They also confirmed the direct interaction between FMRP and Rhoc (Sakano et al., 2017). Another study investigated the role of FMRP in axonal development of the auditory brainstem by inducing FMRP downregulation in avian embryos using CRISPR/Cas9 and shRNA techniques. It resulted in perturbed axonal pathfinding, delay in midline crossing, excess branching of neurites, and axonal targeting errors during the period of circuit development (Wang et al., 2020).

A single study used *Fmr1* knock-out (KO) zebrafish to model the alterations of sensory networks at a cellular level using calcium imaging (Constantin et al., 2020). They identified that the KO larvae had more auditory responsive neurons in the primary auditory regions including the hindbrain and thalamus that were more caudally distributed (Constantin et al., 2020).

Most studies based their work on rodent KO models. The cell size of the KO mice ventral cochlear nucleus (VCN) and the medial nucleus of the trapezoid body (MNTB) was smaller and vesicular GABA transporter protein (VGAT) expression in MNTB was significantly greater than in wild-type (WT) animals (Rotschafer et al., 2015). The same group investigated the developmental phenotypes of *Fmr1* KO mice in the VCN, MNTB, and the lateral superior olive (LSO, Rotschafer and Cramer, 2017). They found that VCN cell size is normal until after hearing onset, while MNTB and LSO show cell size decreases earlier (Rotschafer and Cramer, 2017). VGAT (inhibitory synapse marker) expression was elevated relative to VGLUT (excitatory synapse marker) in KO MNTB before hearing onset (postnatal day 6, Rotschafer and Cramer, 2017). In addition, astrocyte numbers were elevated in KO in VCN and LSO after hearing onset (postnatal day 14, Rotschafer and Cramer, 2017). This means that some phenotypes are observed before auditory activity, while others emerge later, suggesting that FMRP acts at multiple sites and timepoints in auditory system development.

Another study demonstrated the effect of FMRP loss on synaptic rearrangement and function of LSO neurons in adult animals using whole-cell current- and voltage-clamp recordings (Garcia-Pino et al., 2017). KO mice showed a greatly enhanced excitatory synaptic input strength in neurons of LSO, which integrates ipsilateral excitation and contralateral inhibition to compute interaural level differences (Garcia-Pino et al., 2017). In contrast, inhibitory input properties remained unaffected (Garcia-Pino et al., 2017). Moreover, there is an increased number of cochlear nucleus fibers converging onto one LSO neuron. Without any change to individual synapse properties, the immunolabeling of excitatory ending markers revealed an increase in the immunolabeled area, supporting abnormally elevated excitatory input numbers (Garcia-Pino et al., 2017). Due to disturbed development of LSO circuitry, auditory processing was also affected in adult KO mice as shown with single-unit recordings of LSO neurons (Garcia-Pino et al., 2017). Immunofluorescence was used to map the expression of FMRP in the SOC (Ruby et al., 2015). At postnatal day 50, FMRP was widely expressed in neurons of SOC of control rats but not KO. In addition, KO rats had many SOC neurons with a smaller soma and rounder MSO neurons when compared to controls, indicating abnormal neuronal morphology (Ruby et al., 2015), which is similar to what is seen in human post-mortem analysis studies (Kulesza and Mangunay, 2008; Kulesza et al., 2011; Mansour and Kulesza, 2020). There was also a reduction in the expression of glutamic acid decarboxylase (GAD67), a GABA marker, in neurons of the superior paraolivary complex (SPON) and a reduction in the number of calyx terminals associated with neurons of the MNTB (Ruby et al., 2015).

Neural correlates of auditory hypersensitivity in the developing inferior colliculus (IC) in KO mouse were examined using c-Fos immunolabeling and *in vivo* single unit recordings (Nguyen et al., 2020). There was an increase in density of c-Fos neurons in the IC, but not auditory cortex, of KO mice at postnatal day 21 and postnatal day 34 following sound presentation. In addition, *in vivo* single-unit recordings showed that IC neurons of KO mice are hyper responsive to amplitude-modulated tones and tone bursts during development and showed broader frequency tuning curves (Nguyen et al., 2020). KO mice were also used to examine ABRs and quantify excitatory and inhibitory inputs to auditory brainstem nuclei (Rotschafer et al., 2015). The KO showed elevated response thresholds to both click and tone stimuli, ABR amplitudes for early peaks were reduced and the growth of the peak I response with sound intensity was less steep compared with WT (Rotschafer et al., 2015). A study used conditional deletion or expression of *Fmr1* in different cell populations to determine whether *Fmr1* deletion in those cells was sufficient or necessary, respectively, for the audiogenic seizures (AGS) phenotype in male mice. It indicated that *Fmr1* deletion in subcortical glutamatergic neurons that express vesicular glutamate transporter 2 (VGlut2) underlies AGSs (Gonzalez et al., 2019). *Fmr1* deletion in glutamatergic neurons in the IC is necessary for the phenotype, which represents the most precise genetic localization to date for causing AGSs in mice (Gonzalez et al., 2019). It also showed that selective *Fmr1* expression in glutamatergic neurons in an otherwise *Fmr1* KO mouse eliminates AGSs (Gonzalez et al., 2019).

All studies concluded that there were profound differences in *Fmr1* KO auditory brainstem compared to WT. In addition, the studies that focused on the developmental trajectory emphasized its importance (Garcia-Pino et al., 2017; Rotschafer and Cramer, 2017). Identified FMRP target proteins are involved in cellular growth, cellular trafficking, and transmembrane transport (Sakano et al., 2017) and FMRP downregulation resulted in perturbation and errors during the period of circuit development (Wang et al., 2020). Moreover, KO mice had abnormalities in different SOC components (Ruby et al., 2015; Garcia-Pino et al., 2017; Rotschafer and Cramer, 2017). Finally, there's a relationship between KO IC, sound hypersensitivity (Nguyen et al., 2020) and abnormal ABR (Rotschafer et al., 2015).

#### Shank3 Knockout Model

SHANK3 is a gene that encodes the excitatory synapse scaffolding protein SHANK3, and mutations of it have been identified in gene-linkage studies to be associated with ASD (Oberman et al., 2015). Disruptions in SHANK3 domains in mutant mice are associated with behavioral phenotypes and social deficits, but the specific neuronal circuit alterations for the behavioral deficits have not been fully understood (Bariselli et al., 2016). To test whether optogenetic activation of neurons in the dorsal raphe nucleus (DRN) or dopamine neurons in the ventral tegmental area (VTA) may be effective in rescuing the autistic-like social deficits in Shank3 mutant autism mouse model, social training coupled with optogenetic activation of DRN or VTA was performed in KO and WT animals (Luo et al., 2017). The autistic-like social deficits of KO were rescued by social training coupled with optogenetic activation of neurons in the DRN, but not by stimulating dopamine neurons in the VTA, which is a classical reward center (Luo et al., 2017). Another study used shRNA to model Shank3 insufficiency in the VTA of mice (Bariselli et al., 2016). In contrast to the previous study, optogenetic stimulation of the DA neuron in VTA was sufficient to enhance social preference (Bariselli et al., 2016). Additionally, they found that Shank3 downregulation impairs postnatal maturation of metabotropic glutamate receptor 1 (mGluR1) leading to abnormalities in the maturation of excitatory synapses in VTA, driving lifelong synaptic, circuit and behavioral deficits. Systemic treatment with a positive allosteric modulator of mGluR1 during the postnatal period rescued synapse maturation and normalized social deficits in adulthood (Bariselli et al., 2016). The difference in results of DA VTA optogenetic stimulation between the two studies could be due to the mice's age during stimulation and social training. In the second study, mice were between 6 and 7 weeks old when the social preference test was made (Bariselli et al., 2016), whereas age was unfortunately not clearly stated in the first study. However, it is quite possible that there is a critical age period for reversing behavioral impairments.

#### Transgenic (ChR2)-C128S Mutant Mice

To investigate whether the activation of the striatonigral direct pathways is sufficient to induce repetitive behaviors, a study applied optogenetics to activate the substantia nigra pars reticulata (SNr) of a transgenic (ChR2)-C128S mutant mice, which resulted in sustained and chronic repetitive behaviors (Bouchekioua et al., 2018).

#### Ube3a Model

A common and highly penetrant genetic form of ASD results from maternally inherited 15q11-13 triplications that triple the neuron-expressed gene dosage of *UBE3A* (Krishnan et al., 2017). This study showed that increasing *Ube3a* dose in the cell nucleus downregulates the glutamatergic synapse organizer *Cbln1*, which is needed for sociability in mice (Krishnan et al., 2017). It also used a viral vector to activate *Cbln1* in VTA glutamatergic neurons and to reverse the sociability deficits induced by *Ube3a* (Krishnan et al., 2017). Another study examined monoamine levels in *Ube3a* duplicate mice and found that compared to controls, dopamine levels were elevated in *Ube3a* duplicate mice and 5HT levels were decreased in paternal *Ube3a* duplicate mice (Farook et al., 2012).

#### α_7_-nAChR Knockout Model

The encoding gene for the α7-subunit of nicotinic acetylcholine receptor (α7-nAChR) has been associated with ASD (Felix et al., 2019). Using ABRs, a study investigated if α7-nAChR loss of function is associated with abnormal auditory temporal processing (Felix et al., 2019). The KO animals displayed delayed responses with degraded spiking precision. There was a similar delay in responses of neurons in the SPON and ventral nucleus of the lateral lemniscus both of which are thought to shape temporal precision in the midbrain (Felix et al., 2019). The delay in ABR peaks is also found in other animal models (Strata et al., 2005; Scott et al., 2018) and in autistic individuals (Tanguay et al., 1982; Maziade et al., 2000; Rosenhall et al., 2003; Russo et al., 2008; Magliaro et al., 2010; Azouz et al., 2014; Ververi et al., 2015). Moreover, forward masking and gap detection are impaired in KO (Felix et al., 2019), reflecting the forward masking impairment observed in autistic individuals (Källstrand et al., 2010).

#### VPA-Exposed Model

Gestational exposure to valproic acid (VPA), a commonly used anticonvulsant, antiepileptic and mood stabilizer, results in deficits of social behavior in the offspring, modeling ASD symptoms (Dubiel and Kulesza, 2016; Ágota et al., 2020). A study compared developmental neurotoxicity when rats are exposed to VPA at E9 or E11 (Kuwagata et al., 2009). VPA-exposed rats at E11 had abnormal migration of TH-positive and 5-HT neurons, possibly due to the appearance of an abnormally running nerve tract in the pons (Kuwagata et al., 2009). Those observations were more prominent in rats that were shipped pregnant rather than in house bred, which could be due to increased stress (Kuwagata et al., 2009).

Autism is sometimes associated with facial palsy, therefore a study investigated the development of facial nuclei using the VPA-exposed model (Oyabu et al., 2013a). Embryos were exposed to VPA at E9.5 and facial nuclei were analyzed by *in situ* hybridization at E13.5, E14.5, and E15.5 (Oyabu et al., 2013a). The pattern of development was similar between VPA-exposed rats and controls, but the caudal migration of neurons was hindered, and the facial nuclei were smaller in VPA-exposed rats (Oyabu et al., 2013a).

Neuromorphological changes of the dopamine system were studied using the iDISCO method for 3D imaging (Ágota et al., 2020). There was a reduction of mesotelencephalic (MT) axonal fascicles and widening of the MT tract (Ágota et al., 2020). Moreover, there is a reduction of dopaminergic VTA neurons, and tissue level of DA in ventrobasal telencephalic regions but an increase in neuron number in SN (Ágota et al., 2020).

The rostral raphe nucleus (RRN) of E11.5 VPA-exposed rats had narrower neuronal distribution and the whole-embryo had reduced sonic hedgehog expression (Oyabu et al., 2013b). Additionally, another study demonstrated that VPA exposed rats have abnormal 5-HT neuronal differentiation and migration possibly due to distorted patterning of the dorsal raphe nucleus (DRN) and perturbed 5-HT levels postnatally (Miyazaki et al., 2005). Electrical activity of DRN neurons recorded *in vitro* resulted in an increase for VPA-exposed rats (Wang et al., 2018). When examining the mechanism behind the increased excitation/inhibition ratio in synapses, it was found that there was a reduced paired-pulse ratio (PPR) of evoked excitatory postsynaptic currents and increased frequency but unaltered PPR of evoked inhibitory postsynaptic currents (Wang et al., 2018). Therefore, it was concluded that there is an enhanced glutamate but not GABA release (Wang et al., 2018). Moreover, the glutamatergic synaptic transmission was maximized due to occluded spike timing dependent long-term potentiation at the glutamatergic synapses. The intrinsic membrane properties of DRN 5-HT neurons were not altered (Wang et al., 2018).

Neuronal activity in the brainstem circuits was examined using tonotopic maps in VPA-exposed rats via c-Fos expression induced through prolonged auditory stimulation (Dubiel and Kulesza, 2016). More c-Fos neurons were identified with larger dispersion and shifted tonotopic bands in the VPA- exposed rats (Dubiel and Kulesza, 2016). The same group examined the key components of the auditory hindbrain, the ventral cochlear nucleus (VCN) and the SOC of the VPA-exposed rats (Zimmerman et al., 2018). There were significantly fewer and irregularly shaped neurons in both the VCN and the SOC (Zimmerman et al., 2018). Additionally, there was a reduced calbindin and calretinin immunoreactivity and a lower density of dopaminergic terminals (Zimmerman et al., 2018). However, there was no difference in the structure of calyx terminals in the MNTB (Zimmerman et al., 2018). A detailed morphometric analysis of the VPA-exposed SOC concluded MSO and VNTB neurons were smaller and rounder and SPON neurons were smaller, with a different orientation compared to the controls (Lukose et al., 2011). Both MNTB and LSO neurons were larger in VPA-exposed rats and MNTB neurons were generally rounder, while LSO were rounder only in the medial and central limbs (Lukose et al., 2011). Another study examined LL and IC in VPA-exposed rats and found that neurons in the central nucleus of the IC and the dorsal nucleus of the LL were larger than the controls (Mansour et al., 2019). In addition, there were significantly fewer calbindin-immunopositive neurons in the dorsal nucleus of the LL (Mansour et al., 2019). Moreover, VPA exposure resulted in fewer dopaminergic terminals in the central nucleus of the IC (Mansour et al., 2019). Finally, this group examined the proportions of retrogradely labeled neurons in the nuclei of the LL, SOC and CN using stereotaxic injections of the retrograde tracer *Fast Blue* into the central nucleus of the IC (Zimmerman et al., 2020). There were fewer neurons in the auditory brainstem after VPA exposure and fewer neurons that were retrogradely labeled from the central nucleus of the IC (Zimmerman et al., 2020). This indicates altered patterns of input to the auditory midbrain of VPA-exposed rats. Taken together, these results indicate extensive structural and functional abnormalities throughout the auditory brainstem. It is suggested that VPA exposure causes abnormal ascending projections to the IC from both the CN and SOC. Those abnormalities result in difficulties in localization of sound sources and abnormal temporal processing of complex sounds such as vocalizations (Zimmerman et al., 2020).

#### Thalidomide Exposed Model

Exposure to thalidomide (THAL) during the first trimester has been verified as related to the risk of autism in epidemiological studies (Matsuzaki et al., 2012). THAL-exposed rats had a decreased SOC immunoreactivity and smaller MNTB compared to control (Ida-Eto et al., 2017). Another study exposed rats to 16-kHz pure tone auditory stimulus and c-Fos immunostaining (Tsugiyama et al., 2020). THAL rats had an increased number of c-Fos-positive neurons in MNTB compared to the control (Tsugiyama et al., 2020).

A study replicated the results obtained in VPA- exposed rats in THAL-exposed rats, which were a caudal shift of the 5-HT positive neuronal population in the DRN and a decrease in Shh mRNA expression (Miyazaki et al., 2005). Both THAL and VPA had an irreversible effect on the 5-HT neuronal differentiation and migration (Miyazaki et al., 2005). The same model had a decreased SOC immunoreactivity and smaller MNTB compared to control.

#### Cntnap2 Knockout

A loss-of-function mutation in the *CNTNAP2* gene is strongly associated with ASD and language processing deficits therefore one study examined the impact of *Cntnap2* loss on auditory processing, filtering, and reactivity throughout development and young adulthood of rats (Scott et al., 2018). Similar to the atypical ABR responses in autistic individuals mentioned above (Tanguay et al., 1982; Maziade et al., 2000; Rosenhall et al., 2003; Tas et al., 2007; Russo et al., 2008; Magliaro et al., 2010; Azouz et al., 2014; Ververi et al., 2015; Jones et al., 2020; Kamita et al., 2020), hearing thresholds were not altered in KO rats, but there was a reduction in response amplitudes and a delay in response latency of the ABR for juvenile KO animals compared to WT (Scott et al., 2018). The alterations in ABR normalized in adult KO rats indicating a delay in auditory brainstem development (Scott et al., 2018). However, adolescent KO rats displayed deficits in sensory filtering and sensorimotor gating accompanied by increased startle reactivity that persisted into adulthood (Scott et al., 2018).

#### Black and Tan BRachyury T+ Itpr3tf/J (BTBR) Model

The BTBR strain is a phenotypic ASD model which displays behaviors associated with ASD in humans (Chao et al., 2020). BTBR mice have impaired sociability, altered ultrasonic vocalization, and increased self-grooming behaviors which are indicators of impaired sociability, and restricted and repeated behaviors (Chao et al., 2020).

A reduction in TH immunostaining of dopaminergic neurons is observed in the substantia nigra of BTBR mice compared to WT (Chao et al., 2020). They also exhibited decreased expression of striatal dopamine transporter (DAT) and increased spatial coupling between VGLUT1 and TH signals, while no difference is seen in GAD67 (Chao et al., 2020). Additionally, intranasal administration of DA alleviated impairments in non-selective attention, object-based attention, and social approaching (Chao et al., 2020). Taken together, the results indicate a hypofunction of the DA system.

#### Integrin β3 (ITGβ3) Knockout

The *ITG*β*3* gene is associated with both autism and the serotonin system (Ellegood et al., 2012). Volumetric differences between *ITG*β*3* KO and WT mice were examined using high resolution magnetic resonance imaging (Ellegood et al., 2012). There was an 11% reduction in total KO brain volume and a decrease in the lateral wings of the DRN indicating the connection between the *ITG*β*3* gene and the development of the serotonin system (Ellegood et al., 2012).

#### Perinatal Anoxia (PA)

ABRs of rats that underwent PA, an epigenetic factor to autism, and control rats, revealed that PA rats had a delay in all peaks after peak I (Strata et al., 2005). Moreover, interpeak intervals were longer in PA compared to control (Strata et al., 2005). Similar to the ABR alterations in autistic individuals (Tanguay et al., 1982; Maziade et al., 2000; Rosenhall et al., 2003; Tas et al., 2007; Russo et al., 2008; Magliaro et al., 2010; Azouz et al., 2014; Ververi et al., 2015; Jones et al., 2020; Kamita et al., 2020).

#### Radiation Exposure

Prenatal perturbation such as exposure to ionizing radiation or viral infection during early gestation has been linked to neuropsychiatric illnesses including autism. Rhesus macaque monkeys were exposed *in utero* to x-irradiation and allowed to mature to full adulthood (Selemon and Begovic, 2020). Stereological cell counts and soma size measurements of neurons in the SN and VTA revealed a 33% reduction in mean total neuron number in the irradiated monkeys but no difference in soma size between both groups (Selemon and Begovic, 2020).

#### Neuroligin 3 (Nlgn3) Knockout

*Nlgn3* is an ASD-associated synaptic adhesion molecule (Bariselli et al., 2018). VTA DA neuron-specific down-regulation of *Nlgn3* induced aberrant exploration of non-familiar conspecifics as well as deficit in habituation processing (Bariselli et al., 2018). Exploration of a not familiar stimuli is linked with glutamatergic inputs onto VTA DA neurons and an impairment of this novelty-induced synaptic plasticity is seen in in *Nlgn3* KO and *Nlgn3* VTA DA knockdown mice (Bariselli et al., 2018).

#### BALB/c Mice

BALB/c mice express dysregulation in the serotonergic system, therefore two studies examined the role of the serotonergic system in social behaviors that are relevant for ASD (Payet et al., 2018; Russo et al., 2019). In the first study, mice were treated with fluoxetine, a selective serotonin reuptake inhibitor (SSRIs), either acutely or chronically and exposed to the three-chambered social approach test (Payet et al., 2018). Social behavior was decreased by acute fluoxetine, but it increased by chronic fluoxetine compared to controls (Payet et al., 2018). TPH2 enzyme activity was not impacted by SSRI administration, but serotonergic neurons were differentially affected (Payet et al., 2018). The second study showed that BALB/c mice displayed reduced social behavior in three-chamber sociability test and increased anxious behavior in the elevated plus-maze, in combination with decreased 5-HTP accumulation in the rostral and mid-rostrocaudal DRN (Russo et al., 2019).

## Discussion

A final consensus on the morphometric brainstem differences associated with ASD has not been reached, but the most common observation is an alteration in the brainstem size. Studies that observed reduction in either total brainstem volume or at least one of its components (Gaffney et al., 1988; Hashimoto et al., 1989, 1992a,b, 1993a,b, 1995; Herbert et al., 2003; Craig et al., 2007; Jou et al., 2009; Toal et al., 2010; Fredo et al., 2014; Hanaie et al., 2016; Andersson et al., 2020) are more common than studies with any other outcome. Moreover, post-mortem analysis of autistic brains showcased malformation in cells of the olivary complex (Kulesza and Mangunay, 2008; Kulesza et al., 2011; Wegiel et al., 2014).

In accordance with human studies, animal studies also found extensive structural abnormalities throughout the auditory brainstem. VPA-exposed animals have fewer and more dysmorphic VCN (Zimmerman et al., 2018) and SOC (Lukose et al., 2011; Zimmerman et al., 2018) neurons. SOC abnormalities are also seen in the Fmr1 KO model (Ruby et al., 2015; Garcia-Pino et al., 2017; Rotschafer and Cramer, 2017) and THAL-exposed rats (Ida-Eto et al., 2017; Tsugiyama et al., 2020). The morphometric malformation of SOC components in VPA-exposed rats (Lukose et al., 2011) were consistent with malformation in SOC of autistic brains (Kulesza et al., 2011). There are also fewer neurons retrogradely labeled from the central nucleus of the IC (Zimmerman et al., 2020). All this indicates a dysfunction in the ascending projections from the CN and SOC to the IC.

Moreover, the LC is another pons nucleus that is suggested to be atypical in ASD (Granovetter et al., 2019). Autistic individuals experience atypical modulation of LC activity in accordance with changes in attentional demands (Granovetter et al., 2019). Abnormalities in autonomic functioning such as low baseline cardiac parasympathetic activity with evidence of elevated sympathetic tone are seen even in asymptotic autistic individuals (Ming et al., 2005) indicating possible alteration in the brainstem structure that is central in ASD. In addition, studies found a link between brainstem structure and sensory sensitivity (Jou et al., 2009) or motor performance (Travers et al., 2015; Hanaie et al., 2016) in ASD. Taken together, these results suggest that structural aspects of the brainstem, specifically the pons may be related to ASD pathogenies.

The differences of functional measures of the brainstem such as ABR indicate some form of abnormality. A common ABR abnormality is in the peak III amplitude and/or latency (Tanguay et al., 1982; Källstrand et al., 2010; Magliaro et al., 2010; Dabbous, 2012; Ververi et al., 2015; Claesdotter-Knutsson et al., 2019) which is suggested to be an indicator of SOC (Moller, 2006) activity. Additionally, ABR abnormalities are also seen in animal models such as Fmr1 KO mice (Rotschafer et al., 2015), α7-nAChR KO mice (Felix et al., 2019), Cntnap2 KO rats (Scott et al., 2018) and PA (Strata et al., 2005). However, the abnormalities within the animal models are not consistent across different models. Peak I abnormalities are seen in Fmr1 KO mice (Rotschafer et al., 2015) but not observed in Cntnap2 KO rats (Scott et al., 2018) α7-nAChR KO mice (Felix et al., 2019), and PA (Strata et al., 2005). Moreover, animal studies investigating activation of brainstem neurons found a more widespread activation in VPA-exposed rats in response to Dubiel and Kulesza (2016) compared to controls, which suggests that abnormal activation patterns result in altered processing of auditory stimuli.

Brainstem serotonergic system alterations are observed in animal studies using VPA-exposed models (Miyazaki et al., 2005; Kuwagata et al., 2009; Oyabu et al., 2013b; Wang et al., 2018), Thal-exposed model (Miyazaki et al., 2005), ITGβ3 KO (Ellegood et al., 2012) and BALB/c mice (Payet et al., 2018; Russo et al., 2019). Dopaminergic system alterations such as cell morphology and cell count in the SN (Chao et al., 2020; Selemon and Begovic, 2020) or impairment in synaptic plasticity (Bariselli et al., 2018) has been noticed in different animal models including Nlgn3 VTA DA knockdown mice (Bariselli et al., 2018), *in utero* radiation exposed monkeys (Selemon and Begovic, 2020) and BTBR mice (Chao et al., 2020).

To see the effect of age on the brainstem development, we analyzed the age ranges of participants in the studies that reported a negative outcome, meaning no difference between the ASD population and controls. No animal studies reported a negative outcome. However, six human studies showed no differences between the ASD participants and controls (Garber and Ritvo, 1992; Piven et al., 1992; Hardan et al., 2001; Tharpe et al., 2006; Chaddad et al., 2017; Freeman et al., 2018), five of them included adult participants. The total number of studies with only children participants is 31, the number of studies with only adults is 7, the number of studies that included both children and adults is 18, and the remaining studies did not specify age. Four of the negative outcome studies had vast age ranges of 4.45–67.33 years (Freeman et al., 2018), 12.2–51.8 years (Hardan et al., 2001), 8–38 years (Piven et al., 1992), and 18–38 years of age (Garber and Ritvo, 1992), but the means for all of them fell within the adult age range with means m = 19.89 ± 15.34 years, m = 27.7 ± 10.7 years, m = 22.4 ±10.1 years and m = 27.2 ± 5.3 years, respectively. The fifth study included only the mean m=17.01 ± 8.36 years (Chaddad et al., 2017). The sixth study was the only one with only children participants aged 3–10 years (Tharpe et al., 2006). 33.3% of studies with an age mean greater than 18 (*n* = 12) reported a negative outcome while only 5% of studies with an age mean <18 (*n* = 40) reported a negative outcome. Moreover, a post-mortem study that divided the participants into three different groups based on age, 4–8, 11–23, and 29–64 years reported deficits in volume of neuronal nucleus was significant only for the age group 4–8 years (Wegiel et al., 2014).

An animal study that investigated developmental implications using Fmr1 KO showed different abnormalities in SOC morphologies before and after hearing onset (Rotschafer and Cramer, 2017). Moreover, a study using the Cntnap2 KO model observed ABR alterations in juvenile KO rats that normalizes in adulthood, but the deficits in sensory filtering and sensorimotor gating accompanied by increased startle reactivity persisted into adulthood (Scott et al., 2018).

Two longitudinal studies found a difference in the development between autistic and typically developing toddlers. The difference in development between both groups was reduced after a year (Chen et al., 2019; Li et al., 2020). Differences in ABRs (Li et al., 2020) and speech-ABRs (Chen et al., 2019) of autistic and typically developing children were reduced between the two recording sessions that were months apart. This is similar to what is observed in Cntnap2 rat models in which ABR alterations in juvenile KO rats don't persist into adulthood (Scott et al., 2018). Additionally, retrospective studies reported that this delay is observed in newborns that are later diagnosed with ASD (Cohen et al., 2013; Miron et al., 2016).

The results highlight the age effect in autism because of unequal studies with negative outcome in adult populations compared to children populations, and longitudinal studies showing a reduced difference between the ASD and controls as they develop. This warrants both studies with strictly defined and small age ranges for comparison and longitudinal studies that follow up with the same individuals as they develop because a component of the pathogenesis of autism could be a delay in brainstem development. The brainstem holds within it the ascending sensory pathways and a delay in its development could have a cascading effect on the cortex.

## Conclusion

Morphological and structural changes in brainstem size and shape of key brainstem nuclei are observed in both autistic humans (Gaffney et al., 1988; Hashimoto et al., 1989, 1992a,b, 1993a,b, 1995; Herbert et al., 2003; Craig et al., 2007; Kulesza and Mangunay, 2008; Jou et al., 2009; Toal et al., 2010; Kulesza et al., 2011; Fredo et al., 2014; Wegiel et al., 2014; Hanaie et al., 2016; Andersson et al., 2020) and in rodent models of autism (Lukose et al., 2011; Ruby et al., 2015; Garcia-Pino et al., 2017; Ida-Eto et al., 2017; Rotschafer and Cramer, 2017; Zimmerman et al., 2018, 2020; Tsugiyama et al., 2020). Moreover, functional abnormalities are also observed in autistic humans (Tanguay et al., 1982; Ming et al., 2005; Jou et al., 2009; Källstrand et al., 2010; Magliaro et al., 2010; Dabbous, 2012; Travers et al., 2015; Ververi et al., 2015; Hanaie et al., 2016; Claesdotter-Knutsson et al., 2019; Granovetter et al., 2019) and rodent models of autism (Strata et al., 2005; Rotschafer et al., 2015; Scott et al., 2018; Felix et al., 2019). Some studies leveraged the animal models of autism for deeper explorations of serotonergic (Miyazaki et al., 2005; Kuwagata et al., 2009; Ellegood et al., 2012; Oyabu et al., 2013b; Payet et al., 2018; Wang et al., 2018; Russo et al., 2019) and dopaminergic neurotransmitter systems (Chao et al., 2020; Selemon and Begovic, 2020) of which alterations are found in both.

The differences between humans and other species lie in the degree of higher-order cognitive processes, top-down feedback and modulation which consequently gives rise to more complex behaviors in humans compared to other species (Scott et al., 2021). However, the brainstem presents a unique translational opportunity to study the potential mechanisms of disruption in autism since it is highly conserved across species. This translational approach should be exploited using more invasive explorations in animal models to provide answers into the pathogensis of autism. Future studies should aim to investigate the alterations in cellular mechanism that could be the cause of morphological and functional differences seen across species, coupled with human studies on the role of the brainstem in clinical symptomatology of autism that is uniquely human.

## Author's Note

This protocol was registered with the Open Science Framework (osf.io/2hd6m).

## Data Availability Statement

The original contributions presented in the study are included in the article/Supplementary Material, further inquiries can be directed to the corresponding author/s.

## Author Contributions

AS was responsible for the conception, the acquisition, and analysis and interpretation of data for the work. CS and RS were second reviewers for the screening of papers. RS and SS were involved in revising the review paper critically and to ultimately provide approval for publication of the content. All authors contributed to the article and approved the submitted version.

## Funding

AS is funded by Ontario Graduate Scholarship 2021-2022. RS is funded by an NSERC Discovery Grant (RGPIN-2017-04656), a SSHRC Insight Grant (435-2017-0936), the University of Western Ontario Faculty Development Research Fund, the province of Ontario Early Researcher Award, and a Canadian Foundation for Innovation John R. Evans Leaders Fund (37497). Thanks also to the contributions of Western's Brain and Mind Institute and Western's BrainsCAN grant funded by CFREF. We would also like to thank all the quarantining and lock-downs that made this work possible.

## Conflict of Interest

The authors declare that the research was conducted in the absence of any commercial or financial relationships that could be construed as a potential conflict of interest.

## Publisher's Note

All claims expressed in this article are solely those of the authors and do not necessarily represent those of their affiliated organizations, or those of the publisher, the editors and the reviewers. Any product that may be evaluated in this article, or claim that may be made by its manufacturer, is not guaranteed or endorsed by the publisher.

## Supplementary Material

Supplementary Table 1 Summary of all human studies.

Supplementary Table 2 Summary of all animal studies.
