# Supplementary material for: A Systematic Review of Brainstem Contributions to Autism Spectrum Disorder
Source: Front Integr Neurosci. 2021 Nov 1;15:760116. doi: 10.3389/fnint.2021.760116 (PMC8591260; doi:10.3389/fnint.2021.760116)
Supplement: Supplementary Table 1 — Summary of all human studies. [file Table_1.docx]

**Supplementary Table 1 Summary of all human studies. Positive outcomes mean a significant difference between groups is found and a negative outcome means no group difference is found.**

| Study | Participant number | Mean Age  (years) | Outcome | Aim | Summary |
| --- | --- | --- | --- | --- | --- |
| Schmitt et al, 2014 ^a^ | n(ASD) = 65 n(Control) = 43 | 15.2 ±7.7 | positive | To examine attentional processing in ASD through saccadic eye control | Participants made saccades to peripheral targets while having their eye movement recorded using EOG. Autistic participants had reduced accuracy, elevated variability in accuracy across trials, reduced peak velocity and prolonged duration. The saccades took longer to reach peak velocity but had no change in the duration of saccade deceleration. The combination of increased acceleration duration and lowered peak saccade velocity implicate pontine nuclei possibly due to reduced excitatory activity in burst cells that drive saccades relative to inhibitory activity in omnipause cells that maintain stable fixation. |
| Hadjikhani et al, 2017 ^a^ | n(ASD) = 23 n(Control) = 20 | 22.6±1.8 | positive | To examine the effect of constraining gaze to the eye-region on the activation of the subcortical system. | Participants looked at facial emotional stimuli either in free-viewing or restricted to eye-region conditions while having their subcortical activation recording by fMRI. ASD and controls had similar activation patterns in free viewing but ASD group had higher superior colliculus activation in constrained to look in the eyes condition. Additionally, there was a positive correlation between autism symptom severity and subcortical system activation for stimuli of fear and neutral faces, in the free viewing condition. |
| Lai et al, 2015 ^a^ | n(ASD with language delay) = 38  n(ASD without language delay) = 42 n(Control) = 57 | 23.2± 5.6 | positive | To assess the relationship between language development and the neuroanatomy in ASD. | The study included ASD participants with and without language delay and neurotypicals. The neuroanatomy was assessed by MRI images and language development was assed using language history with caregivers based on ADI-R, verbal IQ, word generativity test using the F-A-S task and phonological memory test using the Non-Word Repetition task. Language delay was associated with larger total gray matter volume and larger relative volume at pons and medulla oblongata in adulthood. |
| Freeman et al, 2018 ^c^ | n(ASD) = 22 n(Control) = 22 | 19.89 ± 15.34 | negative | To quantify the density of oxytocin in superior colliculi in postmortem brain tissue from individuals with ASD and typically developing individuals. | There was no significant difference between the oxytocin receptor density in the ASD specimens and the neurotypicals |
| Supekar et al, 2018 ^a^ | n(ASD) = 41 n(Control) = 41 | 10.6 | positive | To examine the structural and functional connectivity of the mesolimbic reward pathway for autistic individuals. | They identified the nucleus accumbens and the VTA white matter tract using high angular resolution diffusion-weighted imaging and functional MRI data and found structural aberrations in these tracts in two cohorts of autistic children. Moreover, they structural aberrations are accompanied by aberrant functional interactions between nucleus accumbens and ventral tegmental area in response to social stimuli. |
| Elvsåshagen et al., 2020^d^ | n(ASD) = 125 n(Control) = 140 | - | positive | To investigate genetic architectures of the brainstem and their involvement in common brain disorders. | Imaging-genetics data was used from a discovery sample of 27,034 individuals and identified 45 brainstem-associated genetic loci, including the first linked to midbrain, pons, and medulla oblongata volumes, and mapped them to 305 genes. Of those genetic loci, 9 were jointly associated with the brainstem volumes and autism. Notably, the shared genetic loci exhibited a mixed pattern of allelic effect such as association with both larger and smaller brainstem volumes. |
| Andersson et al, 2020 ^a^ | n(ASD) = 15 ; n(Control) = 15 | 33 ± 9.1 years | positive | To examine serotonin transporter availability in a sample of autistic adults and correlate regional availability with behavioral phenotypes. | Dynamic PET scan measured the serotonin transporter availability in gray matter. Serotonin transporter availability was found to be significantly lower in total gray matter and the brainstem in ASD group compared to control. Additionally, a general correlation was exhibited between serotonin transporter availability in total gray matter as well as in brainstem and the performance in Reading the Mind in the Eyes test (EYE). |
| Azmitia et al, 2016 ^b^ | n(ASD) = 10 n(Control) = 10 | 14.5 | positive | To compare the process of angiogenesis in post-mortem autistic and control brains | Immunocytochemically labelled post-mortem sections of the brains showed nestin-positive pericytes on blood vessels in all examined brain regions including, midbrain and pons of ASD but not control donors. Measures in the midbrain/pons section were higher in the upper tegmentum and among the fibers of the lower pons for ASD compared to control brains. The labelling pattern of pericytes in consistent with studies of splitting angiogenesis in adult rat brain capillaries where pericytes proliferate. |
| Azouz et al, 2014^e^ | n(ASD) = 30 n(Control) = 15 | 5.4 ± 1.3 | positive | To assess the auditory system in autistic children and to verify the implication of Central auditory processing disorder in autism | ABRs of autistic children were recorded and compared to the normative data collected from 15 normal hearing children. Absolute latency of wave V of the right ear and absolute latency of wave I were significantly prolonged in the ASD group in addition to interpeak latencies (I–V and III–V). |
| Bosco et al, 2018 ^b^ | n(ASD) = 76 n(Control) = 38 | 4.4 ± 1.3 years | positive | To measure the volume and the shape of the brainstem in autistic children | MRI scans were obtained from sedated children and three different data segmentation methods were used. Volume and shape discrepancies were exhibited between segmentation methods, but the brainstem volume was consistently significantly larger in autistic children when compared to controls. |
| Chaddad et al, 2017 ^c^ | n(ASD) = 539  n(Control) = 573 | 17.01±8.36 | negative | To investigate the link between brain regions and clinical variables in MRI | The texture features are derived from MRI scans at multiple resolution levels based on the Laplacian-of-Gaussian filter. Average, standard deviation and entropy quantifier functions are used to summarize texture statistics within automatically segmented neuroanatomical regions. There were no textural feature differences in the brainstem of autistic participants compared to controls. |
| Chen et al, 2019 ^a^ | n(ASD) = 15 n(Control) = 20 | 4.86±1.48 years | positive | To assess the development of auditory brainstem pathway in autistic preschool children using speech-ABR | Speech evoked ABRs recorded twice for each participate with an average of 9.68 months apart. The autistic participants had a prolonged wave V at T1, and a decreased wave E amplitude and prolonged wave F latency at T2. The wave V latency was shortened at T2 compared to T1 for ASD group in addition to a decrease in amplitude of wave A and C. Also, there was a positive partial correlation between the language performance and the wave A amplitude in the ASD group. |
| Claesdotter-Knutsson et al, 2019 ^b^ | n(ASD) = 39  n(Control) = 34 | female 12.71 ± 3.36  male 11.50 ± 3.09 | positive | To explore alterations in auditory processing in the brainstem in autistic youth | Participants were presented with either a forward masked sound or a standard sound while having their ABRs recorded. Wave III had elevated amplitude in ASD group compared to controls with the deviation neuroanatomic region corresponding to the pons. Therefore, the ASD group had more neurons firing in the pons region than the TD group as a response to acoustic stimuli. Additionally, ASD group had higher correlation between the auditory processing of the left and right ears compared to controls which could be related to the difficulties in the processing of everyday sounds in autism. |
| Cohen et al, 2012 ^b^ | n(normal ABR) = 28 n(abnormal ABR) = 46  n(later ASD diagnosis) =14 | neonatal 10.7 ± 15.1 days,4 months and 3.5 ± 1.2 years | positive | To evaluate the contribution of initially abnormal neonatal (ABRs) and 4-month arousal-modulated attention visual preference to later ASD. | The study compared of NICU graduates with normal ABRs to those with initially abnormal ABRs that later resolved. In addition, infants at 4 months post-term age underwent a visual preference task for a random check pattern flashing at 1, 3, or 8 Hz. Outcome measures were PDD Behavior Inventory scores at 3.4 years, and developmental quotients obtained around the same age with the Griffiths Mental Development Scales. 93% of ASD cases were in the abnormal ABR group vs. 56% of non-ASD cases. 28% percent of those in the abnormal ABR group had ASD compared with 4% (one case) in the normal ABR group. Therefore, being in the abnormal ABR group increased the risk of later ASD. There was an association between abnormal ABRs and later reports of repetitive and ritualistic behaviors. Moreover, in abnormal ABR group there was a high correlation between preference for higher rates of stimulation with PDDBI scores, the GMDS Hearing and Speech DQ. |
| Craig et al, 2007 ^a^ | n(ASD) = 14 n(Control) = 19 | 37.9 ±11.4 | positive | To study regional gray-matter and white-matter differences of autistic women | Volumetric magnetic resonance imaging and voxel-based morphometry are used to compare the brain anatomy, regional gray-matter and white-matter, of adult autistic women with controls. Autistic women had a significantly smaller density of white matter in pons. |
| El Shennawy et al, 2014 ^a^ | n(ASD) = 15 n(Control) = 30 | 7.27±1.90 | positive | To assess auditory processing in autism | In speech evoked ABRs, there was a significant difference regarding latencies of waves C and E compared to controls and a highly significant difference for waves D, F, and O. Moreover, the amplitude of control and autistic groups showed a significant difference regarding wave A and a highly significant difference regarding waves C, D, E, F, and O. However, there was no differences regarding click evoked ABRs. |
| Fredo et al, 2014^d^ | |  | positive | To analyze area of subcortical regions using MRI | Used fuzzy Gaussian distribution model-based distance regularized multi-phase level set method to extract subcortical tissue boundaries in MRI scans and measure areas. The brainstem area of autistic individuals was smaller than the controls. |
| Gaffney et al, 1988^d^ | n(ASD) = 13 n(Control) = 35 | 11.3± 4.7 | positive | To study the area of brainstem using MRI scans | The area of the brainstem and its components are calculated from MRI scans. The brainstem area is smaller in the autistic group compared to controls. Additionally, the pontine area is also smaller in the autistic group compared to controls. |
| Garber et al, 1992 ^b^ | n(ASD) = 12 n(Control) = 12 | 27.2 ± 5.3 | negative | To study the area of posterior fossa region in autistic adults using MRI scans | Sagittal MRI scans were used to study the area of posterior fossa components including cerebrum, pons, fourth ventricle and cerebellum. There was no significant difference between the autistic group and the controls. |
| Granovetter et al, 2020 ^a^ | n(ASD) = 23 n(Control) = 24 | 32.04 ± 8.12 | positive | To examine phasic LC activity of autistic individuals under different attentional demands | Phasic pupillary responses are an indication of LC activity. Therefore, as participants performed a simple visual working memory task in the absence or presence of distractor tones their pupil area was recorded. The results indicated that under tightly controlled conditions, task-evoked pupil responses are lower in ASD group than in controls, but only in the presence of task-irrelevant stimuli. This suggests that autistic individuals experience atypical modulation of LC activity in accordance with changes in attentional demands, offering a mechanistic account for attentional atypicality in ASD. |
| Hanaie et al, 2016 ^a^ | n(ASD) = 19 n(Control) = 20 | 9.7±2.5 | positive | To examine the relationship between white matter volume and motor performance in autistic children | MRI scans were processed using a voxel-based morphometry approach to calculate white matter volume. Motor performance was evaluated using the Movement Assessment Battery for Children 2 (M-ABC 2) in which higher scores indicate better motor performance. There was a significant positive correlation between the total test score on the M-ABC 2 and the volume of brainstem white matter. |
| Hardan et al, 2001 ^b^ | n(ASD) = 16 n(Control) = 19 | 22.4 ± 10.1 | negative | To study the volume of posterior fossa region in autistic individuals using MRI scans | Gray and white matter volumes were measured from MRI scans using a semi-automated segmentation process. There were no significant differences in age and total brain volume between the two groups, but full-scale IQ was higher in controls. A decrease in bra |
| Hashimoto et al, 1992a ^b^ | n(ASD) = 12 n(Control-IQ matched) = 15  n(Control-not IQ matched) = 14 | 6.6 ± 1.5 | positive | To study the brainstem area of autistic individuals, individuals with intellectual disability and controls using MRI scans | Two independent clinicians measured the area of posterior fossa region. The participants were autistic individuals with intellectual disability. The brainstem areas of autistic individuals were compared with 2 control groups, one is IQ matched and the other is not. Midbrain and medulla were significantly smaller when compared to non-IQ matched but no difference was observed when compared to IQ matched controls. In addition, the midbrain and medulla to posterior fossa ratio was significantly smaller for autistic individuals compared to controls. |
| Hashimoto et al, 1992b ^c^ | n(ASD- IQ>80) = 10 ; n(ASD- IQ<80) = 19 ; n(Control) = 15 | 4.3 ± 1.6 | positive | to evaluate the brainstem size differences of autistic individuals using MRI scans. | Two independent clinicians measured the area of the brainstem from MRI scans. The intra-rater reliability was> 0.97 and inter-rater reliability was> 0.95. ASD participants are divided into two groups based on IQ either less than 80 or equal/greater than 80. Brainstem width is smaller for ASD group compared to controls and the difference tends to be exacerbated for low functioning. |
| Hashimoto et al, 1993a^b^ | n(ASD) = 12 n(Control) = 24 | 6.1 ± 3.2 | positive | To study the differences in the brainstem and its components in high functioning autism | Two independent clinicians measured the area of the brainstem components from midsagittal MRI scans. The interrater correlations were greater than 0.98. The size of the midbrain and medulla oblongata were significantly smaller than that of the control group, but there was no significant difference between groups with respect to the size of the pons and the entire brain stem area. |
| Hashimoto et al, 1993b ^c^ | n(ASD) = 21 n(Control) = 21 | 4.3 ± 1.7 | positive | To assess the presence of structural changes in the brainstem and cerebellar of autistic children | Two independent clinicians measured the area of the brainstem components from MRI scans. Midbrain, pons, and medulla of autistic children were significantly smaller than control. In the control group, the total brainstem size, and the areas of two components (the midbrain and pons) are significantly correlated with age. However, no relationship was found between midbrain size and age in the autistic group |
| Hashimoto et al, 1989^e^ | n(ASD) = 18 n(Control) = 18 | 3.8 ± 2.2 | positive | To study the differences in the brainstem in autism using MRI scans | Two independent clinicians measured the area of the brainstem from MRI scans. There was a trend of smaller brainstem in autistic individuals compared to controls. |
| Hashimoto et al, 1995^b^ | n(ASD) = 112  n(Control) = 102 | | positive | To investigate the development of the brainstem in a large number of autistic individuals | The autistic individuals and controls were divided into nine age groups (Group 1, 0--< 2 years; Group 2, 2-<4; Group 3, 4--< 6; Group 4, 6--< 8; Group 5, 8--< 10; Group 6, 10-< 12; Group 7, 12-< 14; Group 8, 14-< 16, and Group 9, 16-20). The measurements of brainstem structures from MRI scans were made by 2 independent clinicians. The inter-rater and intra-rater correlations were more than .98. The area of the brainstem and its three components (midbrain, pons, and medulla oblongata) increased with development and revealed a statistically significant correlation coefficient with age for both groups. However, the areas of the brainstem structures in the autistic group were significantly smaller than those in the control group. |
| Herbert et al, 2003 ^a^ | n(ASD) = 17  n(Control) = 15 | | positive | To assess the volume of brain regions including the brainstem of autistic boys using MRI-based volumetric analysis | The total brain was partitioned into its principal regions: cerebrum, brainstem, cerebellum and ventricular system. A primary multivariate general linear model for correlated data was run on all unadjusted regional brain volumes to test for an effect of diagnosis, while controlling for possible effects of age and scanner. The multivariate GLM-CD showed a significant difference between autistic and control children in terms of regional brainstem volume but showed similar proportions when compared to total brain volume and other regional volumes |
| Fredo et al, 2015 ^c^ | n(ASD) = 30  n(Control) = 30 | | positive | To segment and study texture features of subcortical regions in autistic and typically developing brains. | The study used Fuzzy C-means based Augmented Lagrangian multiphase level set method to segment the subcortical regions in MRI scans and calculated the texture features such as energy and entropy for them. The mean entropy values obtained from the subcortical regions are higher in autistic subjects compared to controls. The entropy values and energy values obtained from the total brain and brainstem vary significantly between ASD group and control group. The delay in subcortical region development is associated with higher entropy values. |
| Jones et al, 2020 ^a^ | n(ASD) = 18 n(Control) = 18 | 2.9 ± 0.45 | positive | To examine the auditory processing differences of autistic and typically developing toddlers. | Auditory click and speech evoked responses were recorded for autistic toddlers and typically developing toddlers. The results indicate a prolonged click Wave I–V interpeak latency, click Wave III–V interpeak latency and speech /da/ Wave O latency for the autistic toddlers compared to controls. However, there was no significant relationships between any behavioral measure and measure of auditory processing. |
| Jou et al, 2008 ^a^ | n(ASD) = 22 n(Control) = 22 | 10.7 ± 1.4 | positive | To examine white matter and gray matter differences in brainstem of autistic children. | Gray and white matter volumes were measured from MRI scans using a semi-automated segmentation process. There were no significant differences in age and total brain volume between the two groups, but full-scale IQ was higher in controls. A decrease in brainstem gray-matter volume was observed in the autism group before and after controlling for TBV. No significant differences were observed in white-matter volume. A significant relationship was observed between brainstem gray-matter volume and oral sensory sensitivity as measured by the Sensory Profile Questionnaire. |
| Jou et al, 2013 ^a^ | n(ASD) = 23 n(Control) = 23 | 10.6 ± 1.4 | positive | To investigate longitudinal volumetric differences in brainstem of autistic children. | Two sets of MRI brain scans were obtained with a 2-year time interval. Autistic and typically developing youth showed different patterns of growth in whole brainstem volumes. Whole brainstem volume remained relatively stable in typically developing. In contrast, autistic individuals showed increases with age reaching volumes comparable to controls by age 15 years. The brainstem increase was driven mainly by gray matter. |
| Källstrand et al, 2010 ^c^ | n(ASD) = 15 n(Control) = 15 | 38.4±10.8 | positive | To examine ABR forward masking patterns of individuals with Asperger syndrome compared to control subjects. | A square-shaped click pulse was used as a probe for both the control ABR condition and the auditory forward masking setup. In the forward masking paradigm, the square-shaped click pulse is preceded by a masker. ABR waveforms in the forward masking condition showed that wave III amplitudes were significantly lower in AS individuals than in the control groups. |
| Kamita et al, 2019 ^a^ | n(ASD) = 15 n(Control) = 15 | 9.07 ±1.75 | positive | To analyze the neural encoding of verbal and nonverbal stimuli in individuals with autism spectrum disorder using ABRs | Click and speech evoked ABRs were measured, and the results indicated that click evoked interpeak interval III---V in the ASD group was greater and speech evoked wave V was shorter for ASD group than the typically developing group. |
| kulesza et al, 2011^b^ | n(ASD) = 9 n(Control) = 4 | 15 ± 4 | positive | To examine neuronal morphology and neuronal number in human superior olivary complex | There was a significant alteration in cell body morphology and decreased neuronal number in the SOC of the autistic brainstem. In ASD brains, the medial superior olive (MSO) was the most severely and consistently malformed of the SOC nuclei. MSO neurons were less in number, much smaller (area) and rounder in autistic brains. The lateral superior olive (LSO) contained many more round/oval neurons and LSO neurons were smaller, and more round in ASD group but similar in orientation to controls. Moreover, there was a reduction in neuron number in the LSO of ASD group. Medial nucleus of the trapezoid body (MNTB) neurons were of different proportions: oval to fusiform to stellate ratios. Also, there is a reduction in the number of MNTB and they were less round and of different orientation in the ASD group compared to controls. Superior paraolivary nucleus neurons were less and rounder in the ASD group. Ventral nucleus of the trapezoid body neurons were rounder in ASD group. Lateral nucleus of the trapezoid body neurons were less, smaller and rounder in the ASD group. |
| kulesza et al, 2008^b^ | n(ASD) = 5  n(Control) = 2 | | positive | To examine the superior olivary complex SOC in post-mortem brain tissue from autistic individuals. | MSO neurons in the autistic brains were smaller in terms of cell body area, perimeter and major axis and these neurons were rounder than in control. Moreover, comparing the angle measurements indicated that the orientation of MSO neurons in the ASD group was more heterogeneous than control. |
| Li et al, 2020 ^a^ | n(ASD) = 20 n(Control) = 20 | T1: 3.40 ± 0.26  T2 : 4.40 ± 0.26 | positive | To analyze the developmental auditory brainstem pattern in preschool autistic children | Click evoked ABRs were collected at two time periods (T1 and T2) that were a year apart. There was no significant change between the 2 recording sessions in TD group. However, in ASD group, peak V latency was significantly shortened at T2 compared to that recorded at T1. The interpeak latency of peaks I–V was shorter at T2 compared to T1 in the autistic children. Compared to the TD group, ASD was associated with longer latencies for peak V and longer interpeak latencies of I-III, I–V at T1. In addition, ASD group also indicated longer latencies of peak III and peak V, longer interpeak latencies of I-III and I–V at T2 compared to the TD group. |
| Magliaro et al, 2010 ^b^ | n(ASD) = 16 n(Control) = 25 | 11.94 | positive | To examine the auditory processing of autistic individuals through electrophysiological assessments | Clicked evoked ABRs, middle latency response and cognitive potential (P300) were recorded for each participant. There was a significant prolongation in latencies of waves III and V and interpeaks I-III and IV of autistic participants compared to controls. |
| Mansour et al, 2020 ^b^ | n(ASD) = 7  n(Control) = 3 | 7.14 | positive | To investigate number and morphology of SOC neurons in ASD | Brainstem sections of the SOC nuclei were 3D volume rendered using Amira software. In ASD subjects, the MSO, LSO and SPON occupied a significantly smaller volume compared to controls. MNTB and LNTB were smaller in ASD group compared to controls. The overall volume of the SOC is consistent across brain weights and changes in the size of the SOC and constituent nuclei in ASD are not likely attributable to changes in total brain weight. |
| Maziade et al, 2000 ^b^ | n(ASD) = 75 n(ASD relatives) = 251; n(Control) = 521 | 7.3 ± 5.1 | positive | To investigate inheritable ABR differences in autistic probands and their unaffected relatives | Click-evoked ABRs were recorded for autistic probands, their relatives and controls. The relatives group included first-, second- and third-degree relatives. Autistic proband had prolongation of inter-peak latency I-III. Furthermore, the same I-III inter-peak latency prolongation is observed in the unaffected first-degree relatives of the autistic probands compared with controls. |
| Ming et al, 2005 ^b^ | n(ASD symptomatic ) = 15 n(ASD asymptomatic) = 13 n(Control) = 17 | 9.4± 4.9 | positive | To measure baseline cardiovascular autonomic function in children with autism using the NeuroScope | NeuroScope is a device that can measure brainstem function in real-time. Resting cardiac vagal tone (CVT), cardiac sensitivity to baroreflex (CSB), mean arterial blood pressure (MAP), diastolic blood pressure (DBP), systolic blood pressure (SBP) and heart rate (HR) were recorded for an autistic group that experienced or signs of autonomic dysfunction, and autistic group that did not exhibit symptoms or signs of autonomic dysfunction and a control group. The CVT and CSB were lower in combination with a elevation in HR, MAP and DBP in all autistic children compared to controls. Additionally, the levels of CVT and CSB were lower in the symptomatic group than in the asymptomatic group. The levels of CVT and CSB were not related to age in all the three groups. These results suggest that there is low baseline cardiac parasympathetic activity with evidence of elevated sympathetic tone in children with autism regardless if they have symptoms or signs of autonomic dysfunction. |
| Ming et al, 2016 ^b^ | n(ASD) = 19 n(Control) = 18 | 9.1 ± 4.5 | positive | To evaluate the cardiorespiratory functions in ASD to including the breathing pattern | Autonomic function was monitored at a defined resting state using the NeuroScope. The non-invasive real time beat-to-beat blood pressure was measured and fed into the NeuroScope where HR, CVT and cardiac sensitivity to baroreceptor were derived from the ECG and blood pressure waveforms. Respiratory rate and rhythm were measured simultaneously, and respiration was analyzed breath by breath. Various respiratory dysrhythmias, particularly Biot’s and Cheyne–Stokes respiration, were detected in autistic children in combination with greater variability in respiratory rhythm and amplitudes than controls. The respiratory dysrhythmia in autistic children was associated with a lower cardiac vagal activity. The Biot’s breathing and Cheyne–Stokes respiration coupled with cardiac vagal hypofunction in ASD suggests a brainstem dysfunction. |
| Miron et al, 2016 ^b^ | n(ASD infants ) = 30 n(ASD toddlers) = 40  n(Infant Control) = 30 | 1.61 ± 0.82 months or 2.38 ± 0.5 years | positive | To examined whether prolonged ABR latencies appear in infancy | Wave V was significantly prolonged in those who later developed ASD as compared with controls. Classification using this measure enabled accurate identification of ASD infants with 80% specificity and 70% sensitivity. Autistic toddlers had prolonged absolute and interpeak latencies compared to clinical norms. |
| Miron et al, 2020 ^a^ | n(ASD) = 321 n(Control) = 138,844 | 1.76 ± 3.08 days | positive | To examine whether newborns who later develop ASD show early ABR abnormalities | Newborn ABR data was extracted from their Universal Newborn Hearing Screening, including newborns who were later diagnosed with ASD. Autistic newborns had prolongations of their ABR phase and V-negative latency compared with controls. Autistic newborns also exhibited greater variance in their latencies compared to previous studies in older autistic individuals, likely due in part to the low intensity of the ABR stimulus. |
| Piven et al, 1992^d^ | n(ASD ) = 14 n(Control IQ matched) = 14  n(Control SES matched) = 15 | 27.7 ± 10.7 | negative | To investigate pons and fourth ventricle area using MRI | Measurements were made by two blinded raters. After correcting for midsagittal brain area, there was no significant difference in the size of the pons in autistic subjects and the controls in groups I (nonverbal IQ matched) and Il (socioeconomic status matched). |
| Ramezani et al, 2018 ^a^ | n(ASD) = 28 n(Control) = 28 | 14.36 ± 1.86 | positive | To investigate the subcortical speech processing in high functioning autistic children | Speech evoked ABRs were recorded. Latencies of all waves in s-ABRs and duration of V-A complex were longer in autistic children compared with controls. |
| Rosenhall et al, 2003 ^c^ | n(ASD, boys ) = 75 n(ASD, girls) = 26 n(Control) = 59 | boys: 8.1 girls: 9.2 | positive | To study the cochlear nerve and the auditory brainstem pathway in autistic children | Click-evoked ABRs were recorded and ABR analysis was done separately for boys and girls. The III-V interpeak latency and waves I and V absolute latencies were prolonged for autistic boys and girls compared with controls. |
| Russo et al, 2008 ^b^ | n(ASD) = 28 n(Control) = 28 | 9.9 ± 1.92 | positive | To examine the subcortical representations of prosodic speech in autistic children | Speech evoked ABRs were recorded with descending and ascending pitch contours conditions. A subgroup of the autistic children showed deficient pitch tracking evidenced by increased frequency and slope errors and reduced phase locking compared with controls. |
| Russo et al, 2009 ^b^ | n(ASD) = 21  n(Control) = 21 | 9.9 ± 1.92 | positive | To examine the processing and transcription of speech sounds at a brainstem level | Speech evoked ABRs were measured in a quiet and with background noise conditions. Autistic children exhibited deficits in both the neural synchrony (timing) and phase locking (frequency encoding) of speech sounds, despite normal click-evoked brainstem responses. They also exhibited reduced magnitude and fidelity of speech-evoked responses and increased degradation of responses by background noise in comparison to controls. |
| Suzuki et al, 2013 ^b^ | n(ASD) = 20  n(Control) = 20 | 23.3 ± 4.0 | positive | To identify brain regions associated with excessively activated microglia in the whole brain | Participants underwent PET scans with microglia radiotracer. The binding potential values were higher in multiple brain regions in autistic young adults compared with controls. Brain regions with increased binding potentials included the cerebellum, midbrain, pons, fusiform gyri, and the anterior cingulate and orbitofrontal cortices. The pattern of binding was similar between both groups, but the magnitude of the binding potential was greater for the autistic participants. |
| Tanguay et al, 1981^d^ | n(ASD) = 14  n(Control) = 14 | 5.2 | positive | To investigate ABR differences in infantile autism | Children underwent audiometry click evoked ABR testing. Autistic children had prolonged peak I latency for right ear stimulation and prolonged peak III latency for left ear stimulation compared to controls. |
| Tas et al, 2007^C^ | n(ASD) = 30  n(Control) = 15 | 3.8 ± 1.3 | positive | To evaluate hearing using transient evoked otoacoustic emission (TEOAE) and ABRs | Participants underwent ABR and TEOAE testing. For ABR, the mean III–V interpeak latencies in both ears of autistic children were longer than controls. For TEOAE, there was significant differences between both groups. |
| Tharpe et al, 2006 ^a^ | n(ASD) = 12  n(Control) = 12 | 5.58 | negative | To describe the auditory characteristics of autistic | Participants underwent ABR recording but no significant between-group differences for absolute or interpeak latencies were observed. |
| Toal et al, 2010 ^a^ | n(ASD ) = 65  n(AS) = 39 n(Control) = 33 | 30 ± 8 | positive | To examine if localized differences in brain anatomy are associated with variation in the clinical phenotype | Voxel-based morphometry was used to investigate brain volume using MRI scans. White-matter reductions were present bilaterally in the brainstem for autistic participants compared to controls. When only participants with Asperger syndrome were compared to controls, there was gray-matter reductions bilaterally in the brainstem. |
| Travers et al, 2015 ^a^ | n(ASD) = 43 n(Control) = 43 | 4 ± 1 | positive | To investigate if atypical white matter microstructure in the brain mediated the relationship between motor skills and ASD symptom severity | Fractional anisotropy of the brainstem’s corticospinal tract predicted both grip strength and autism symptom severity and mediated the relationship between the two. These findings suggest that brainstem white matter may contribute to autism symptoms and grip strength in ASD. |
| Ververi et al, 2015 ^a^ | n(ASD) = 14 n(Control) = 14 | 15.1 ± 6.96 | positive | To examine ABR deficits among autistic subjects | Autistic subjects had prolonged absolute latencies I, V and III, and shortening of interpeak latency I-V compared to controls. In addition, abnormalities were more common among autistic participants. |
| Wegiel et al, 2015 ^a^ | n(ASD) = 76  n(Control) = 44 | | positive | To establish the course of neuronal nuclear and cytoplasmic volume changes throughout the lifespan of autistic individuals | Cytoplasm and nucleus of neurons in post-mortem autistic brains were stereologically analyzed. Deficits in volume of neuronal nucleus was significant only for age group 4 - 8 years. In addition, the trajectory of neuronal nucleus volume in ASD was opposite to the control trajectory. The autistic cohort had a significant increase in older groups while a distinctive feature of control was a decrease in neuronal nucleus volumes in both older groups. |

^a^ quality score of 10

^b^ quality score of 9

^c^ quality score of 8

^d^ quality score of 7

^e^quality score of 6
