# Supplementary material for: A Systematic Review of Brainstem Contributions to Autism Spectrum Disorder
Source: Front Integr Neurosci. 2021 Nov 1;15:760116. doi: 10.3389/fnint.2021.760116 (PMC8591260; doi:10.3389/fnint.2021.760116)
Supplement: Supplementary Table 2 — Summary of all animal studies. [file Table_2.docx]

**Supplementary Table 2 Summary of all animal studies.**

| Animal Studies | | | |
| --- | --- | --- | --- |
| Study | Animal model | Aim | Summary |
| Wang et al, 2020 ^a^ | *FMRP* KO chicken embryo | To study the role of FMRP in embryonic development of the auditory hindbrain | FMRP was downregulated in the avian embryos using CRISPR/Cas9 and shRNA techniques which resulted in perturbed axonal pathfinding, delay in midline crossing, excess branching of neurites, and axonal targeting errors during the period of circuit development. |
| Sakano et al, 2017^b^ | White Leghorn chick hatchlings | To explore the molecular role of FMRP in the avian nucleus laminaris (NL) which is a brainstem nucleus | 94 potential FMRP targets were identified in the NL using proteomic analysis. These proteins are enriched in pathways involved in cellular growth, cellular trafficking and transmembrane transport. Moreover, the dendritic localization of several proteins in NL was verified and the direct interaction of FMRP with one candidate, RhoC, by in vitro RNA binding assays was confirmed. |
| Rotschafer et al, 2015 ^c^ | *Fmr1* KO mice | To investigate ABRs and quantify excitatory and inhibitory inputs to auditory brainstem nuclei | The KO cell size of the VCN and MNTB was smaller and VGAT expression in MNTB was greater compared to WT. Moreover, the KO had increased response thresholds to both click and tone stimuli, ABR amplitudes for early peaks were reduced and the growth of the peak I response was less steep compared with WT. |
| Rotschafer et al, 2017 ^a^ | *Fmr1* KO mice | To describe the auditory phenotypes during development of Fmr1 knockout | Before hearing onset, MNTB and LSO cell sizes were smaller and VGAT (inhibitory synapse marker) expression was elevated relative to VGLUT (excitatory synapse marker) in MNTB of KO compared to controls. After hearing onset, VCN cell is smaller in KO and astrocyte numbers were elevated in VCN and LSO of KO. |
| Nguyen et al, 2020 ^a^ | *Fmr1* KO mice | To investigate neural correlates of auditory hypersensitivity in the developing inferior colliculus (IC) of Fmr1 KO | The density of c-Fos neurons increased in the IC, but not auditory cortex, of KO mice at postnatal day 21 and 34 after sound presentation. In addition, in vivo single-unit recordings showed that IC neurons of KO mice are hyper responsive to amplitude-modulated tones and tone bursts during development and showed broader frequency tuning curves. |
| Garcia-Pino et al, 2017 ^a^ | *Fmr1* KO mice | To examine the effect of FMRP on synaptic rearrangement | Fmr1KO mice showed enhanced excitatory synaptic input strength in neurons of LSO which integrate ipsilateral excitation and contralateral inhibition to compute interaural level differences. In contrast, inhibitory input properties remained unaffected. Due to the disturbed development of LSO circuitry, auditory processing was also affected in adult Fmr1KO mice as shown with single-unit recordings of LSO neurons. |
| Ruby et al, 2015 ^a^ | *Fmr1* KO mice | To map the expression of FMRP in SOC | FMRP was widely expressed in SOC neurons WT but not KO. SOC of Fmr1 KO had a smaller soma and rounder MSO neurons than WT, indicating abnormal neuronal morphology. GABA marker, GAD67, was lower in neurons of the SPON of KO and a lower number of calyx terminals associated with neurons of the MNTB than WT. |
| Gonzalez et al, 2019 ^a^ | *Fmr1* KO mice | To determine the locus in which Fmr1 deletion causes AGSs | Conditional deletion or expression of Fmr1 in different cell populations resulted in pinpointing that Fmr1 deletion in subcortical glutamatergic neurons that express VGlut2 underlies AGSs. Fmr1 deletion in glutamatergic neurons in the IC is necessary for the phenotype. Selective Fmr1 expression in glutamatergic neurons in an otherwise Fmr1 KO mouse eliminates AGSs. |
| Luo et al, 2017 ^a^ | *Shank3* knockout mice | To rescue the autistic-like social deficits in Shank3 mutant autism mouse model | Social training was coupled with optogenetic activation of DRN or VTA. The autistic-like social deficits of KO were rescued by the DRN coupling, but not by stimulating dopamine neurons in the VTA. |
| Bariselli et al, 2016 ^a^ | *Shank3* knockout mice | To investigate SHANK3 role in maturation of social reward circuits in the VTA | ShRNA is used to model Shank3 downregulation in the VTA of mice which impaired postnatal maturation of mGluR1 leading to abnormalities in the maturation of excitatory synapses in VTA. Systemic treatment with a positive allosteric modulator of mGluR1 during the postnatal period rescued synapse maturation and normalized social deficits in adulthood. Additionally, optogenetic stimulation of DA neuron in VTA of KO was sufficient to enhance social preference. |
| Bouchekioua et al, 2018 ^d^ | Transgenic (ChR2)-C128S mutant mice | To investigate if activation of the striatonigral direct pathways is sufficient to induce repetitive behaviors | Optogenetics was used to induce excitation of D1 receptor-expressing mediums spiny neurons in the SNr of a Transgenic (ChR2)-C128S mutant mice which resulted in sustained and chronic repetitive behaviors. |
| Krishnan et al, 2017 ^a^ | transgenic *Ube3a* mice | To explore the pathway in which an increase in Ube3a gene impacts sociability | The increasing of UBE3A in the nucleus downregulates Cbln1, glutamatergic synapse organizer, which is needed for sociability in mice. A viral vector that activated Cbln1 in VTA glutamatergic neurons reversed the sociability deficits induced by Ube3a. |
| Farook et al., 2012 ^c^ | *Ube3a* duplication mice | To determine the effect of Ube3a duplication on monoamine levels in different brain regions. | Dopamine levels were elevated in Ube3a duplicates compared to controls and 5HT levels were decreased only in paternal Ube3a duplication animals but remained unaffected in maternal Ube3a duplication animals. |
| Felix et al, 2019 ^b^ | *α7-nAChR* KO mice | To investigate the impact of α_7-_nAChR loss on auditory temporal processing | The KO had delayed responses with degraded spiking precision. There was a similar delay in responses of neurons in the SPON and ventral nucleus of the LL both of which are thought to shape temporal precision in the midbrain. Moreover, forward masking and gap detection which are temporal acuity measures were impaired in KO. |
| Agota et al, 2020 ^a^ | VPA-exposed mice | To study the quantitative morphometric parameters of the midbrain dopaminergic centers and pathways, and the concentration of DA | Neuromorphological changes of the dopamine system were studied using 3D imaging with full transparency. There was a reduction of mesotelencephalic axonal fascicles and widening of its tract. Moreover, there is a reduction of DA VTA neurons, and tissue level of DA in ventrobasal telencephalic regions but an increase in neuron number in SN. |
| Dubiel et al, 2016 ^c^ | VPA-exposed rats | It studied neuronal activity in brainstem circuits and irregular tonotopic maps in VPA-exposed rats. | Neuronal activation was examined using immunohistochemistry for c-Fos after exposure to 4- or 16-kHz tones. VPA-exposed rats had larger dispersion of c-Fos neurons and a shifted tonotopic bands compared to controls which suggests hyper-responsiveness to sounds and disrupted mapping of sound frequencies. |
| Mansour et al, 2019 ^c^ | VPA-exposed rats | To examine LL and IC in VPA-exposed rats | Nuclei of the LL and the central nucleus of the IC were examined using histochemistry, morphometric techniques, and immunofluorescence. Neurons were larger in the central nucleus of the IC and the dorsal nucleus of the LL, fewer calbindin-immunopositive neurons in the dorsal nucleus of the LL, and fewer dopaminergic terminals in the central nucleus of the IC of VPA-exposed rats compared to controls. |
| Zimmerman et al, 2018 ^c^ | VPA-exposed rats | To examine the ventral cochlear nucleus (VCN) and SOC of the VPA-exposed rats | Examination of LL and IC of VPA-exposed rats found fewer and differentially shaped neurons in both the VCN and SOC, reduced calbindin and calretinin immunoreactivity, and a lower density of dopaminergic terminals compared to controls. However, there was no difference in the structure of calyx terminals in the MNTB. |
| Zimmerman et al, 2020 ^c^ | VPA-exposed rats | To examine the axonal projection patterns of brainstem nuclei to the IC in VPA-exposed animals | Examination of retrogradely labeled neurons in the nuclei of the LL, SOC and CN revealed fewer neurons in the auditory brainstem and fewer neurons that were retrogradely labeled from the central nucleus of the IC after VPA exposure indicating altered patterns of input to the auditory midbrain. |
| Lukose et al, 2011 ^d^ | VPA-exposed rats | To examine SOC of VPA exposed rats | For VPA exposed rats, MSO and VNTB neurons were smaller and rounder and SPON neurons were smaller with a different orientation compared with controls. Both MNTB and LSO neurons were larger, and MNTB neurons were generally rounder while LSO were rounder only in the medial and central limbs for VPA exposed rats. There were fewer neurons in SOC in VPA exposed rats. |
| Oyabu et al, 2013a ^b^ | VPA-exposed rats | To investigate the migration of facial neurons and initial facial nucleus formation | Both expressed the same pattern of development, but VPA-exposed rats had hindered caudal neuron migration and smaller facial nuclei. |
| Oyabu et al., 2013b ^c^ | VPA-exposed rats | To examine prenatal development of 5-HT neurons | Whole-embryo in situ hybridization at E11.5 showed reduced sonic hedgehog expression in VPA-exposed rats. Additionally, whole-mount immunohistochemistry of the hindbrain and quantitative analysis of 5-HT neurons in the rostral raphe nucleus (RRN) revealed that narrower neuronal distribution in the caudal part of the RRN. |
| Kuwagata et al, 2009 ^b^ | VPA-exposed rats | To observe neurotoxicity of the fetal brain shortly after VPA exposure | Developmental neurotoxicity was compared in exposed to VPA at E9 or E11. VPA exposed rats at E11 had abnormal migration of TH-positive and 5-HT neurons, possibly due to the appearance of an abnormally running nerve tract in the pons. Those observation were more prominent in rats that were shipped pregnant rather than in-house bred. This could be due to increased stress. |
| Wang et al, 2018 ^a^ | VPA-exposed rats | To examine the function of DRN 5-HT neurons | Increase in electrical activity and excitation/inhibition ratio in synapses of DRN neurons for VPA-exposed rats. It is due to a reduced paired-pulse ratio (PPR) of evoked excitatory postsynaptic currents and increased frequency but unaltered PPR of evoked inhibitory postsynaptic currents which means that there is an enhanced glutamate but not GABA release. Moreover, the glutamatergic synaptic transmission was maximized due to occluded spike timing dependent long-term potentiation at its synapses. Finally, the intrinsic membrane properties of DRN 5-HT neurons were not altered. |
| Miyazaki et al, 2005 ^a^ | VPA or THAL exposed rats | To examine whether serotonergic neuronal differentiation and migration is altered in two animal models of autism | THAL/VPA exposure caused a shift of the 5-HT positive neuronal population caudally in the DRN and a decrease in Shh mRNA expression. Therefore, THAL and VPA had an irreversible effect on the 5-HT neuronal differentiation and migration which may result from distorted patterning of the DRN and perturbed 5-HT levels postnatally. |
| Ida-Eto et al, 2017 ^d^ | THAL exposed rats | To investigate whether the auditory brain center is affected in THAL exposed rats | On P50, THAL exposed rats had a decreased SOC immunoreactivity and smaller MNTB compared to control. |
| Tsugiyama et al, 2019 ^a^ | THAL exposed rats | To investigated whether abnormal response occurs in the brainstem following sound stimulus | Rats were exposed to 16-kHz pure tone auditory stimulus and c-Fos immunostaining. THAL rats had increased number of c-Fos positive neurons in MNTB compared to the control. |
| Constantin et al, 2020 ^a^ | *Fmr1* knockout zebrafish | To model at a cellular level the brain wide alterations of sensory networks in Fmr1 KO zebrafish | Calcium imaging was used to model the alterations of sensory networks by recording from the entire brain at a cellular resolution. There was no difference in response to visual stimuli but there were differences in auditory processing. KO larvae had more auditory responsive neurons in the primary auditory regions including the hindbrain and an increased inter-regional connectivity at lower sound intensities compared to WT. |
| Chao et al, 2020 ^a^ | BTBR KO mice  *Fmr1* KO mice | To define the alteration in DA pathways and whether DA can be utilized as a potential therapeutic agent using a genetic and phenotypic animal model of ASD | A reduction in TH immunostaining of DA neurons is observed in the SN of BTBR mice compared to WT. However, Fmr1-KO animals displayed abnormal morphology of TH-positive axons in the striatum. Both strains exhibited decreased expression of striatal DA transporter and increased spatial coupling between VGLUT1 and TH signals, while no difference is seen in GAD67. Intranasal DA administration rescued social deficits in both strains. |
| Ellegood et al, 2012 ^a^ | *ITGβ3* Knockout | To examine the volumetric differences between ITGβ3 KO and WT | Volumetric differences between ITGβ3 KO and WT were examined using MRI. There was an 11% reduction in total KO brain volume and a decrease in the lateral wings of the DRN indicating a link between the ITGβ3 gene and the development of the serotonin system. |
| Strata et al, 2005 ^d^ | Perinatal anoxia rats | To explore the effect of perinatal anoxia on auditory behavior and on auditory system | PA rats had an increase in acoustic thresholds and reduction in processing efficiencies in the auditory behavior task. All ABR peaks of PA were delayed except peak I and interpeak intervals were longer in PA compared to control. |
| Selemon et al, 2020 ^a^ | Fetal Radiation Exposure of Non-human Primate | To examine the impact of altering neurogenesis in early gestation on neuron number in the SN and VTA | Rhesus macaque monkeys were exposed in utero to x-irradiation on E39 to E41 and allowed to mature to full adulthood. Stereologic cell counts and somal size measurements of neurons in the midbrain specifically in the SN and VTA revealed a 33% reduction in mean total neuron number in the irradiated monkeys but no difference in soma size between both groups. |
| Bariselli et al, 2018 ^a^ | *Nlgn3* KO | To investigate the involvement of DA neurons in specific aspects of sociability such as the response to and the preference for non-familiar | Nlgn3 is an ASD associated synaptic adhesion molecule. VTA DA neuron-specific down-regulation of Nlgn3 induced aberrant exploration of non-familiar conspecifics as well as deficit in habituation. Exploration of nonfamiliar stimuli is linked with glutamatergic inputs onto VTA DA neurons and an impairment of this novelty-induced synaptic plasticity is seen in in Nlgn3KO and Nlgn3VTA DA knockdown mice. |
| Payet et al, 2018 ^b^ | BALB/c mice | To examine the role of serotonergic systems in social behaviors that are relevant for ASD | Mice were treated with fluoxetine a selective serotonin reuptake inhibitors (SSRIs) either acutely or chronically and exposed to the three-chambered social approach test. Social behavior was decreased by acute fluoxetine, but it increased by chronic fluoxetine compared to controls. TPH2 enzyme activity didn’t get impacted by SSRI administration, but serotonergic neurons were differentially affected. |
| Scott et al, 2018 ^a^ | *Cntnap2* KO rats | To test the impact of Cntnap2 loss on auditory processing, filtering, and reactivity throughout development and young adulthood | Hearing thresholds were not altered in KO but there was a reduction in response amplitudes and a delay in response latency of the ABR for juvenile KO compared to WT. The alterations in ABR normalized in adult KO indicating a delay in auditory brainstem development. Adolescent KO displayed deficits in sensory filtering and sensorimotor gating accompanied by increased startle reactivity that persisted into adulthood. |
| A. M. Russo et al., 2019 ^a^ | BALB/c mice | To examine the role of serotonergic systems in social behaviors that are relevant for ASD | BALB/c mice displayed reduced social behavior and increased anxious behavior in combination with decreased 5-HTP accumulation in the rostral and mid-rostrocaudal DRN. |

^a^ quality score of 10

^b^ quality score of 9

^c^ quality score of 8

^d^ quality score of 7
